# CENP-A nucleosome clusters form rosette-like structures around HJURP during G1

**DOI:** 10.1038/s41467-019-12383-3

**Published:** 2019-09-30

**Authors:** Leonid Andronov, Khalid Ouararhni, Isabelle Stoll, Bruno P. Klaholz, Ali Hamiche

**Affiliations:** 1 Centre for Integrative Biology (CBI), Department of Integrated Structural Biology, IGBMC, CNRS, Inserm, Université de Strasbourg, 1 rue Laurent Fries, 67404 Illkirch, France; 2 Institute of Genetics and of Molecular and Cellular Biology (IGBMC), 1 rue Laurent Fries, Illkirch, France; 3 Centre National de la Recherche Scientifique (CNRS), UMR 7104 Illkirch, France; 4 Institut National de la Santé et de la Recherche Médicale (Inserm), U964 Illkirch, France; 5 Université de Strasbourg, Illkirch, France; 6 Department of Functional Genomics and Cancer, IGBMC, CNRS, Inserm, Université de Strasbourg, 1 rue Laurent Fries, 67404 Illkirch, France

**Keywords:** 3-D reconstruction, Fluorescence imaging, Centromeres

## Abstract

CENP-A is an essential histone H3 variant that epigenetically marks the centromeric region of chromosomes. Here we show that CENP-A nucleosomes form characteristic clusters during the G1 phase of the cell cycle. 2D and 3D super-resolution microscopy and segmentation analysis reveal that these clusters encompass a globular rosette-like structure, which evolves into a more compact structure in late G1. The rosette-like clusters contain numerous CENP-A molecules and form a large cellular structure of ∼250–300 nm diameter with remarkably similar shapes for each centromere. Co-localization analysis shows that HJURP, the CENP-A chaperone, is located in the center of the rosette and serves as a nucleation point. The discovery of an HJURP-mediated CENP-A nucleation in human cells and its structural description provide important insights into the mechanism of CENP-A deposition and the organization of CENP-A chromatin in the centromeric region.

## Abstract (web summary)

Human centromeric nucleosomes contain the specific CENP-A histone variant, which replaces canonical histone H3 and epigenetically marks the centromeres. Here the authors show that CENP-A nucleosomes form large supramolecular clusters during the G1 phase of the cell cycle which are arranged as rosette-like structures.

## Introduction

The centromere is the chromosomal locus required for kinetochore formation that ensures chromosome segregation. Human centromere DNA comprises 171 bp AT-rich alpha-satellite monomers that are arranged into tandem arrays^1,2^. Both the size (≤ 4 Mb) and repetitive nature of human centromeres have impeded the assembly of molecular maps and limited comprehensive functional and structural analyses^1^. Centromere DNA sequences are highly divergent between species but eukaryotic centromeres have in common a specialized nucleosome containing centromeric specific histone 3 variant CENH3 (CENP-A in mammals), which replaces canonical histone H3^3–8^. CENP-A synthesis and deposition at centromeres is cell cycle dependent. In human, the peak of CENP-A synthesis occurs during G2-phase^9^ and deposition of CENP-A at centromeric DNA starts late in mitosis and continues to early G1 phase^9,10^. The deposition of CENP-A is mediated by the histone chaperone HJURP, which co-localizes with CENP-A at the centromere in early G1 phase^11–13^. The N-terminal domain of HJURP interacts with the CENP-A targeting domain (CATD) and mediates its deposition to centromeres^13^. The uncoupling of CENP-A deposition from replication^14^ on centromeric DNA results in “dilution” of CENP-A at centromeres of daughter chromosomes. This raises the question how CENP-A gets distributed equally to the daughter centromeres^15,16^. An interesting aspect is that the “dilution” of CENP-A in daughter centromeres during S phase and its subsequent restoration at the next G1 phase may be required for faithful cell division. In this context, a key question is how CENP-A is specifically delivered to centromeric chromatin and maintained on centromeres during the cell cycle. In addition, it remains poorly understood how the HJURP complex, carrying newly synthesized CENP-A, is specifically recruited to centromeres. A recent study, using synthetic human artificial chromosomes, stresses the importance of alpha-satellite DNA transcription for HJURP recruitment and centromeric CENP-A assembly^17^. The centromeric chromatin may adopt a specific structure at G1 to guide the HJURP/CENP-A complex to centromeres. In this context, shedding light on the 3D organization of centromeres at G1 is of fundamental importance.

Recent studies focused on the structure of the centromere chromatin at the single nucleosome level using reconstituted particles^18,19^. However, little is known about the in vitro 3D organization of CENP-A nucleosome arrays^20^, and the in vivo structure of the specialized CENP-A chromatin that ultimately recruits kinetochore proteins remains highly elusive. Immunofluorescence imaging of human CENP-A typically displays every centromere as a diffraction-limited spot at any point of the cell cycle^21^, but much higher resolution imaging would be required to address the underlying fine structure. Previous light and electron microscopy studies have given interesting insights into the kinetochore organization during mitosis^22–25^. However, the fine structure of the in situ centromeric chromatin in interphase has never been visualized.

In this work, we set out to study the spatial distribution of CENP-A in the cell nucleus using super-resolution microscopy, including single-molecule localization microscopy (SMLM), a technique that makes it possible to obtain fluorescence images with a resolution of 20–50 nm^26^. Direct stochastic optical reconstruction microscopy (dSTORM^27^), photo-activated localization microscopy (PALM^28^) and also stimulated emission depletion (STED^29^) imaging reveal that the CENP-A/HJURP nucleosome complexes form characteristic clusters at interphase. Time point analysis shows that they have a very specific organization that evolves during the cell cycle. Indeed, we find that CENP-A nucleosome clusters progress from a rosette-like shape in early G1 phase to a filled spherical shape in late G1. HJURP, the CENP-A chaperone, is located within the cavity in the center of the centromeres at the beginning of G1. This specific organization of the G1 centromeric chromatin suggests a special role of HJURP to nucleate CENP-A deposition and the assembly of centromeric chromatin. The 3D reconstruction of these CENP-A clusters provides a structural description of centromere nucleation and overall architecture.

## Results

### CENP-A nucleosomes form clusters in the centromere region

**Fig. 1:**
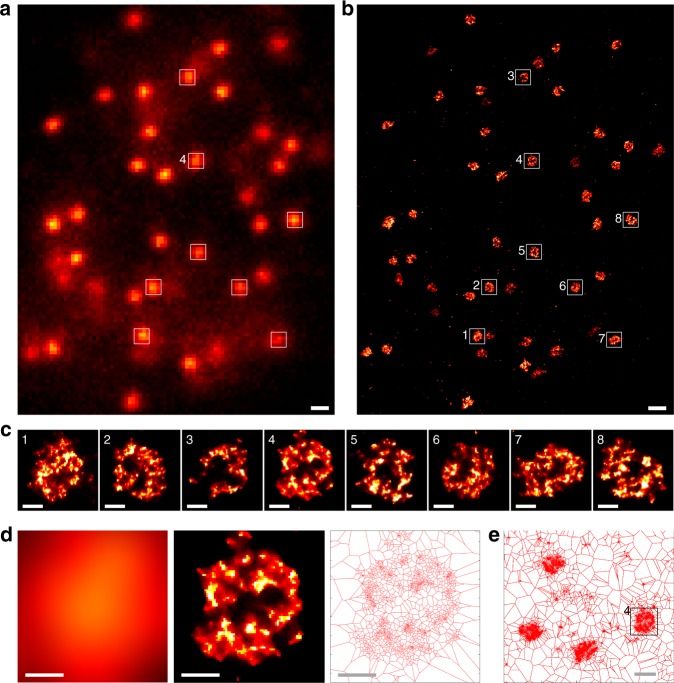
SMLM images of centromeres in a non-synchronized U2OS cell. **a** Conventional epifluorescence image of CENP-A. **b** High-resolution SMLM image of the same region revealing that CENP-A in fact forms characteristic clusters. **c** Zoomed view of several centromeres from this cell. **d** Cluster #4 imaged in classical epifluorescence microscopy (left), SMLM (center) and represented as a Voronoi diagram built on localizations of fluorophores (right). **e** Larger region of the Voronoi diagram containing cluster #4 and other clusters. A significant portion of the non-synchronized cells have centromeres of this rosette-like shape, suggesting that they are formed in a particular phase of the cell cycle. Scale bars, 500 nm (**a**, **b**, **e**) and 100 nm (**c**, **d**); for similar experiments in HeLa cells see Supplementary Figs. 1 and 2

To analyze centromeric chromatin by super-resolution imaging, we have specifically visualized the CENP-A nucleosomes by using an anti-CENP-A antibody coupled to Alexa Fluor conjugated secondary antibodies. We first imaged non-synchronized U2OS and HeLa cells. Interestingly, CENP-A nucleosomes form large clusters in the centromere region of every chromosome of a given cell (Fig. 1 and Supplementary Figs. 1–2). These clusters exhibit a globule-like structure of ∼250–300 nm diameter.

**Fig. 2:**
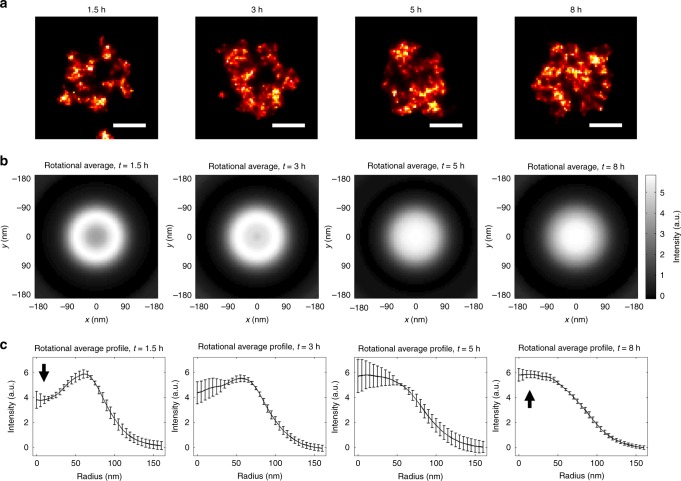
Statistical analysis of CENP-A clusters in synchronized U2OS cells. **a** Manually picked representative clusters for each of the time points (1.5, 3, 5, and 8 h, shown on top); scale bars: 100 nm. **b** Rotationally averaged images of the sum of many aligned clusters for every time point. **c** Profiles of the rotationally averaged images shown in (**b**) with the standard deviation displayed with error bars. For this analysis, SMLM images of all centromeres from five (1.5–5 h) or 6 (8 h) different cells were used: in total 211 clusters for the 1.5 h time point, 196 clusters for 3 h, 243 clusters for 5 h, and 214 clusters for 8 h. The error bars indicate the standard deviation between the rotationally averaged images of the sum of the CENP-A clusters of each analyzed cell for the given time point. The analysis shows that the density distribution progresses from a shell to a more compact sphere shape exhibiting a dip at the beginning of the radial profile at 1.5 h (arrow) and a maximum at 8 h (arrow); see Source Data file

Next, in order to analyze the behavior of these clusters throughout the cell cycle we imaged synchronized U2OS cells. We focused on the early G1 phase, because at this stage centromeric chromatin undergoes a major change due to the deposition of the newly synthesized CENP-A. For this we performed a time point analysis and imaged the G1 phase of the cell cycle. Reconstructed SMLM images show a major change in the shape of CENP-A clusters upon the progress of the cells in G1 phase (Fig. 2). At the beginning of the cell cycle, until ~3 h after mitosis, the centromeres are organized in extensively structured clusters (contrasted clusters with sharp edges, often composed of several small sub-clusters) with a cavity in the middle. As G1 proceeds (~5 h after mitosis), the clusters adopt a less structured cloud-like shape with more diffuse edges and a more compact central region, while the central hole disappears. To endorse these findings statistically, we performed an in-depth image analysis. For this, we picked the CENP-A cluster images from 2D SMLM images of cells fixed at 1.5, 3, 5, and 8 h after mitosis, and centered and iteratively aligned them to their rotationally averaged sum (see Methods). This analysis shows that a structural transformation of the centromeric chromatin occurs from a shell-shaped hollow cavity to a filled shape at the beginning of G1 phase (Fig. 2). Over time, the total number of CENP-A molecules increases as expected due to their deposition in early G1 (Supplementary Fig. 3), while the diameter of the clusters slightly decreases (from 235 to 188 nm on average between time points 1.5 and 8 h) indicating a general compaction of the structure (i.e., ~50% in 3D; Supplementary Fig. 4). In order to exclude that the hollow structure of CENP-A clusters is seen due to a poor penetration of antibodies inside potentially dense centromere chromatin, we imaged CENP-A, tagged with the mEOS2 photo-convertible protein, using stably transfected U2OS cells. PALM imaging of mEOS2 reveals the same hollow shape of CENP-A clusters in early G1 (Supplementary Fig. 5), confirming the above results obtained using immunofluorescence super-resolution imaging.

### Analysis of the CENP-A clusters by 3D SMLM and segmentation

**Fig. 3:**
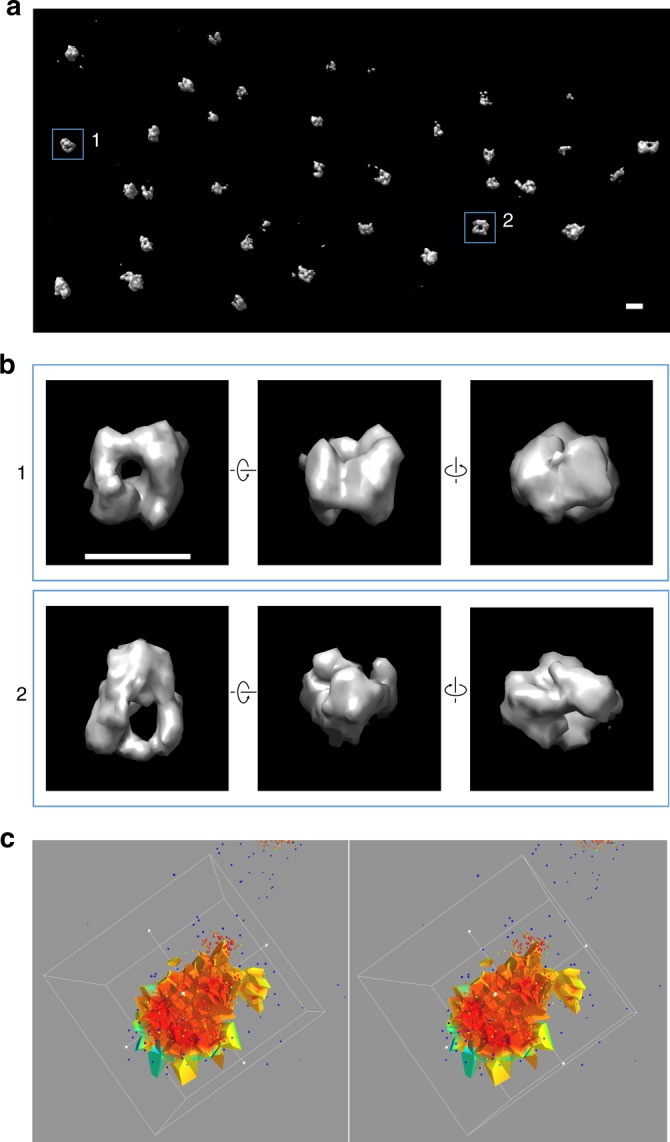
3D SMLM imaging of CENP-A in a U2OS cell fixed at 1.5 h after mitosis. **a** Characteristic 3D rosette-like clusters can be seen. The image is created as a 3D Voronoi-based density map^43^ and displayed with the Chimera software^51^; scale bar: 300 nm. **b** Zoomed-in images display rotated views of particles, revealing a cavity in the center formed by a CENP-A shell; scale bar: 300 nm. **c** A 3D Voronoi diagram built on 3D SMLM data of CENP-A (stereo representation); view cut through a cluster; color coding from high (red) to low fluorophore densities (green). See also Supplementary Fig. 6 for a 3D reconstruction obtained from 2D images that also shows the rosette-like structure

To investigate in more detail the 3D structure of centromeric chromatin, we then performed 3D SMLM based on astigmatism^30^ using adaptive optics^31^. 3D SMLM imaging in combination with our recently developed Voronoi-diagram 3D segmentation method^32^ shows that the CENP-A clusters adopt a characteristic rosette-like three-dimensional structure which is remarkably similar in all chromosomes of a given cell (Fig. 3). Consistent with these data, and thanks to the similar size of the clusters (see size analysis in Supplementary Fig. 4), a 3D reconstruction was also obtained by back-projection and averaging of 2D projection images of different CENP-A clusters of a cell. The 3D reconstruction obtained from angular reconstitution (a technique we imported from the field of electron microscopy, see Methods) shows the same typical rosette structure (Supplementary Fig. 6).

### CENP-A clusters nucleate on the histone chaperone HJURP

**Fig. 4:**
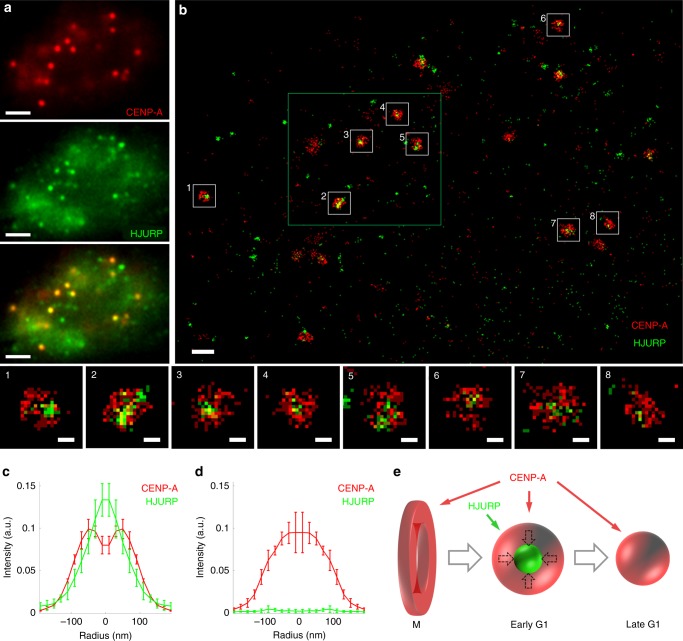
SMLM imaging of CENP-A with its chaperone HJURP (U2OS cells). **a** Wide-field images of CENP-A and HJURP show their overall co-localization at centromeres. **b** SMLM imaging demonstrates that CENP-A (red) and HJURP (green) form clusters of different shapes in which CENP-A and HJURP do not co-localize at the individual molecule level. Bottom left panels 1–8: magnified centromeres from image (**b**) demonstrate that an HJURP cluster is primarily situated in the empty center of a CENP-A cluster. The dSTORM images (panels **b** and 1–8) are reconstructed in the histogram mode with a bin size of 20 nm. The green rectangle in panel (**b**) indicates the region that is zoomed in and displayed as a Voronoi diagram in Supplementary Fig. 7. Scale bars, 2 µm (**a**), 500 nm (**b**), and 100 nm (1–8); for similar experiments using STED imaging see Supplementary Fig. 8. **c**, **d** Radial profiles of the rotationally averaged images of the CENP-A and HJURP particles at 1.5 h (**c**) and 5 h (**d**) after mitosis, using five cells for each time point; see Source Data file. **e** Schematic description of the evolution of the shape of CENP-A clusters during the cell cycle

Having identified a major reorganization/maturation of CENP-A clusters during G1, we next analyzed the distribution of HJURP within these clusters using co-localization SMLM. Surprisingly, while at low-resolution HJURP appears to be superimposed on CENP-A (Fig. 4a) as also seen by classical confocal microscopy^12^, at high-resolution HJURP is distinctly resolved and is found located within a smaller cluster that fills the cavity of the CENP-A clusters until 3 h after mitosis (Fig. 4b and Supplementary Fig. 7). In addition, we performed STED microscopy on synchronized U2OS cells, which fully corroborates our above findings about the centromeric chromatin organization. This includes both the shape of the clusters and the localization of HJURP in the middle of the CENP-A clusters at the beginning of the G1 phase (Supplementary Fig. 8).

## Discussion

The mechanism of CENP-A deposition during the G1 phase of the cell cycle has been a long-standing question. Here, we used super-resolution fluorescence microscopy to study the 3D organization of centromeres concomitant to CENP-A deposition, revealing that at interphase CENP-A forms well-defined clusters within the centromeric regions of chromatin of human cells. The existence of CENP-A clusters was found by extensive dSTORM imaging in 2D and even in 3D (Figs. 1–4) and was also independently confirmed by STED imaging (Supplementary Fig. 8). The analysis at different G1 time points (Fig. 2) identifies specific structural changes of these clusters that are associated with the concomitant deposition of newly synthesized CENP-A, as we show below. Quantification using Voronoi-based clustering (Fig. 3 and Supplementary Fig. 2) and PALM imaging (Supplementary Fig. 5) shows that the clusters contain numerous CENP-A molecules; these were also localized individually by dSTORM imaging, consistent with previous data using stochastic fluctuation analysis^33^. Unlike in mitosis, where CENP-A forms a disk-like structure corresponding to the base of the kinetochore^24^ (Supplementary Fig. 9), in early G1, the CENP-A clusters organize into a characteristic three-dimensional rosette-like structure (Figs. 1–3 and Supplementary Figs. 1, 2, 5, 6), with HJURP being located in the center of the ring/shell as revealed from co-localization analysis (Fig. 4 and Supplementary Figs. 7–8). As G1 progresses (~5 h after mitosis) these clusters adopt less structured cloud-like shapes with more diffuse edges, and the central cavity is no longer seen but instead becomes a more compact CENP-A structure (Fig. 2). Our results show and also quantify that there is a clear structural transition in the centromeric chromatin organization upon CENP-A deposition, not only at the level of individual nucleosomes, but also at the level of the higher-order chromatin structure in the range of ~100–300 nm. In early G1 (1.5–3 h after mitosis) this structure accumulates considerable amounts of HJURP in its central cavity. The accumulation of HJURP coincides with the timing of CENP-A deposition suggesting that deposition of CENP-A occurs inside the rosette where HJURP serves as a nucleation point. Interestingly, newly deposited CENP-A forms clusters at time point 7 h that have a shape similar to that of the pre-existing old CENP-A (Supplementary Fig. 10). This suggests that the filling of the cavity where HJURP is located goes along with a global reorganization of the ring. In fact, analysis of the localizations using a stable CENP-A-mEOS2 cell line reveals a significant increase from 1.5 to 7 h after mitosis (~1.5 times), which is consistent with the idea that the CENP-A concentration should double during G1 (Supplementary Fig. 3; taking into account that some new CENP-A is being deposited already before the first time point). While CENP-A is being uploaded a general compaction of the structure occurs. Moreover, centromeric CENP-A organization in mitosis displays elongated rod- or ring-like structures (Supplementary Fig. 9), which are clearly distinct from the globular rosette-like (shell-like) structure in early G1, during which CENP-A loading nucleates on HJURP. These data show that the CENP-A organization is very dynamic and consists of an evolution from a disk-like structure in mitosis to a globular rosette-like structure in G1, which appears to be bi-partite comprising two joined hand-like halves (Figs. 1 and 3). The rod or disk-like structure known during mitosis thus appears to evolve to a ring-like rosette structure, which becomes more compact and spherical/globular during late G1 (Fig. 4e).

Strikingly, the general features of the rosette-like structure, which we suggest to call “assemblosome”, with an outer diameter of 250–300 nm and an inner diameter of ~100 nm, are conserved among all chromosomes in the imaged human and mouse cell lines (U2OS, HeLa and MEF cells; Supplementary Figs. 1, 2, 11). This points to a conserved functional role of this supramolecular structure. This large rosette could theoretically accommodate several thousand nucleosomes per centromere. However, due to its compacted nature and the optical resolution restricted to ~30 nm, it is unclear whether this rosette structure is composed only of CENP-A nucleosomes or whether it contains interspersed CENP-A and other histone H3 variant nucleosomes (Supplementary Fig. 12). Previous work analyzing extended centromeric chromatin fibers in human and Drosophila revealed that centromeres are formed by an interruption of CENH3-positive nucleosome clusters by clusters missing CENH3^34,35^. Given the size of the rosette we cannot exclude the possibility that this structure is also interspersed by non-CENP-A nucleosomes.

Homologs of both CENP-A and HJURP are found in all eukaryotes, from fission yeast to humans. This suggests that the newly discovered specific rosette-like structure of the HJURP-centromeric nucleosome complexes is probably a common feature of eukaryotic cells at the G1 phase of the cell cycle. At present the function of this assemblosome is unknown. We speculate that this remarkably large cellular structure facilitates the targeting and the deposition of CENP-A in the centromeres and that it is essential for the assembly of functional centromeric chromatin. The discovery of the assemblosome and its present structural description opens the possibility to analyze centromeric CENP-A deposition in more detail, in particular to address the regulatory mechanism of HJURP-mediated nucleation of CENP-A clusters that triggers the assembly of the entire kinetochore.

## Methods

### Cell culture and immunofluorescence

U2OS cells (ATCC, HTB-96), HeLa cells (ATCC, CCL-2.2), and MEF cells (derived from 13 days C57BL/6 mouse embryos) were synchronized using the Thymidine-Nocodazole synchronization. Briefly, the cells were plated in P150 petri dishes and 24 h later the medium was replaced with prewarmed complete Dulbecco’s modified Eagle’s medium (DMEM) supplemented with 2.5 mM Thymidine for 18 h. The cells were washed with complete DMEM and 100 ng/ml Nocodazole was added for 16 h to block cells in mitosis. Floating mitotic cells were then collected, washed twice with complete DMEM, resuspended in complete DMEM and plated in glass-bottom petri dishes (MatTek Corporation P35G-0.170-14-C). Cells were collected after 1.5, 3, 5, and/or 8 h for immunofluorescence experiments. DNA and microtubule labeling confirm as expected that sister chromatids and microtubules are not seen in the nucleus during early G1 (Supplementary Figs. 13 and 14).

For immunofluorescence, cells were washed twice in PBS and fixed in 4% paraformaldehyde in PBS for 20 min at room temperature, washed twice in PBS, washed twice in PBS with 0.1 M Glycine pH 8.5 and once in PBS. Cells were then permeabilized with 0.05% Triton X-100 in PBS for 15 min, washed twice in PBS, saturated with PBS with 1% bovine serum albumin (PBB) for 1 h, incubated with primary antibody (Supplementary Table 1) diluted in PBB for 1 h, washed three times in PBB, incubated with secondary antibody (Supplementary Table 1) diluted in PBB for 1 h, washed twice with PBB and twice with PBS. For DNA visualization, the YoYo-1 dye dilution was 1:10,000.

For transient transfection, cells were plated in P150 petri dishes; 24 h later they were transfected with calcium phosphate with 20 µg of plasmid and incubated for 40 h. For generation of the stable cell line expressing mEOS2-CENP-A, the Phoenix A cells were plated in 6-well plates and incubated for 5 h. Then they were transfected with calcium phosphate with 10 µg of plasmid and incubated for 24 h, then washed briefly and incubated for 24 h. U2OS cells were infected with the viral particles (retrovirus) and incubated for 48 h. They were then selected with magnetic beads Dynabeads CD4 (ThermoFisher Scientific, 11145D) and incubated for 72 h. After that, the U2OS cells were selected two more times with the Dynabeads magnetic beads.

For transfection in phase S, U2OS cells were first plated in P150 dishes and incubated for 24 h. They were treated with Thymidine 2.5 mM for 18 h, washed twice with complete DMEM and released for 3 h in complete DMEM. Being synchronized in S phase, the cells were transiently transfected with calcium phosphate. After 5 h, the cells were treated with Nocodazole 100 ng/ml for 16 h, blocking them in mitosis. Floating cells were collected, washed twice in complete DMEM, resuspended in complete DMEM and plated in glass-bottom petri dishes (MatTek P35G-0.170-14-C). Cell were collected after 1.5 h for early G1 and 7 h for late G1. Imaging was performed at time point 7 h only because the signal was too weak at time point 1.5 h.

SMLM was performed using the imaging buffers described below and in Supplementary Table 2.

1. Vectashied/TDE, adapted from ref. ^36^. 20% v/v Vectashield (Vector Laboratories H-1000) mixed with 70% v/v 2,2′-thiodiethanol (TDE, Sigma-Aldrich 166782) in PBS. The refractive index of this buffer is 1.49^37^. Mounting was performed by incubating the sample in PBS solutions with gradually increased concentrations of TDE (10% v/v, 25% v/v, and 50% v/v), for 10 min each.
2. 10 mM Cysteamine (MEA, Sigma-Aldrich 30070) in PBS, pH adjusted to 7.5 with 25 mM HEPES.
3. OxEA^38^. 50 mM Cysteamine hydrochloride (Sigma-Aldrich 30080), 3% v/v OxyFluor^TM^ (Sigma-Aldrich SAE0059) and 20% v/v sodium DL-lactate solution (Sigma-Aldrich L1375) in PBS, pH adjusted to 8.5 with NaOH.

The samples for STED microscopy were mounted in ProLong^TM^ Diamond Antifade Mountant (ThermoFisher Scientific P36961).

### Super-resolution imaging

The SMLM imaging was performed on in-house Leica SR GSD system (a dSTORM/PALM microscope; for a detailed protocol see also ref. ^39^). We used the HCX PL APO 160 ×/1.43 Oil CORR TIRF PIFOC objective that provides an equivalent pixel size of 100 nm on the camera. The camera, the lasers and the stage were the same as on the Leica SR GSD 3D system. For 3D SMLM, the astigmatism was induced with a MicAO 3DSR adaptive optics system installed between the microscope stage and the camera (the value of the astigmatism was set to 0.2 µm root mean square). The deformation of the PSF was calibrated by defocusing the objective with a step of 50 nm and imaging Tetraspeck fluorescent beads with diameter of 200 nm. The strength of astigmatism was adjusted so to obtain a more isotropic resolution.

The acquisition was started manually after observing first single-fluorophore events (blinking) during illumination with the appropriate laser. The experimental parameters of SMLM experiments were optimized for each fluorophore and mounting medium. The exposure time of a frame was 7–25 ms; the electron multiplying gain of the camera was 300; the laser power during the acquisition was 25–100%. After a few minutes, as the number of blinking events dropped, the sample started to be illuminated additionally by a 405 nm laser with gradual increase of its intensity in order to facilitate the single-molecule return into the ground state. The acquisition was stopped after almost complete bleaching of the fluorophore. For counting of the relative number of CENP-A localizations (Supplementary Fig. 3), each cell was imaged with exactly the same experimental parameters. For DNA visualization with YoYo-1 dye, blinking was not as strong, which may explain the lower resolution obtained compared with Alexa dyes; however, showing chromatin decondensation (Supplementary Fig. 13) does not require high resolution.

STED microscopy was done on a Leica TCS SP8 STED 3X microscope with the HC PL APO CS2 100×/1.40 OIL objective. The fluorophores were excited with a supercontinuum laser at 15% of its maximal power. Alexa Fluor 488 was depleted with a 592 nm continuous-wave laser set at 70–90% of its maximal power. Alexa Fluor 555 was depleted with a 660 nm continuous-wave laser set at 75% of its maximal power. The fluorescence was detected with the HyD detectors working in the photon counting mode with time gating, detecting light from 0.5 to 6.0 ns after the excitation laser pulse.

### Data processing

The localization and fitting of single-molecule events were performed in real time during acquisitions in Leica LAS AF software with the “direct fit” fitting method. The localization tables were then exported for further processing in the SharpViSu software workflow^40^ and with customized Matlab procedures. The drift was detected and corrected in two or three dimensions, for 2D and 3D data, respectively, using cross-correlation-based approach. Briefly, the data set was divided on several consecutive subsets, from each of them a histogram image with pixelation of 20 nm was built, and the shift between these images was detected with subpixel precision and interpolated linearly throughout intermediate frames. The shift value was then subtracted from the coordinates of every frame. The lateral drift was detected using the projection on the XY plane, and the axial drift (for 3D data) was detected using the average shift, calculated from the XZ and YZ projections. The procedure was repeated iteratively several times assuring absence of detectable residual drift. Chromatic aberrations were first calibrated using 0.2 µm TetraSpeck™ microspheres, the shift between the channels was interpolated with a 2nd order polynomial function and was subtracted from the localizations of one of the imaging channels^40^. To reduce the number of localizations of the same fluorophore and improve localization precision the data were processed by averaging the coordinates of consecutive events around each localization using the following procedure: for each original localization *N* on frame *n*, localizations within a distance of 50 nm from *N* on frame *n* + 1, were searched for. If found, the search continued for the frame *n* + 2, etc. The search stopped when there was no localization on the following frame. All the found localizations were merged into a single one, which was assigned to the frame *n*, its coordinates were set as the average of the coordinates of all the consecutive localizations and its photon count was set as the sum of the photon counts^40^. FRC_1/7_ resolution^41,42^ was calculated in SharpViSu^40^, using the corrected data in histogram representation with a pixel size of 10 nm and 90 points of spatial frequency. Imaging experiments with CENP-A alone and with HJURP in synchronized cells were repeated several times using new seed cells each time (e.g., results presented in Figs. 2 and 4 are from different cells, different antibodies and image acquisitions but give the same distribution for CENP-A). Dual-color imaging of CENP-A and HJURP with inverted secondary antibodies gave the same results. Supplementary Table 2 summarizes the quality parameters of the corrected datasets.

SMLM images were built as 2D or 3D density maps based on the Voronoi diagrams method^37,43,44^ (Figs. 1–3, Supplementary Figs. 1 and 5), or as 2D histograms (Fig. 4, Supplementary Figs. 9–14). For the analysis of the CENP-A localization (Fig. 2), the CENP-A clusters were picked from the images using the Boxer tool from the EMAN2 package^45^. These images of the clusters were then normalized (division by standard deviation of the pixel values) and iteratively aligned to their rotationally averaged sum using the IMAGIC software^46^, a procedure similar to that applied previously for structural studies of the nuclear pore complex^47^. The resulting aligned particles were again rotationally averaged and their radial profile was calculated in Matlab (Fig. 2); this uses the reconstructed super-resolution images (after drift-correction). For statistical evaluation of these data, all aligned particles originating from every individually analyzed cell were first rotationally averaged and summed separately for each cell. Radial profiles were calculated for each of these average images (one per cell). The standard deviation shown in Fig. 2c in error bars was calculated for every point of these radial profiles. The half-radius of CENP-A clusters was calculated as the radius where the cumulative radial profile of the clusters reaches 50%. This gives exactly half the radius for disks with constant density and sharp edges and less than half the radius for particles whose density decreases at their periphery, which is the case for the CENP-A clusters.

For the analysis of the localization of CENP-A detected with mEOS2 (Supplementary Figs. 3 and 5) and the localization of HJURP within the CENP-A clusters (Fig. 4), the analysis was performed using a full localization-based approach. After correction of drift and chromatic aberrations, the localization data were used for reconstruction of images in the histogram representation with a pixel size of 20 nm. The approximate positions of the centers of the CENP-A clusters were detected either automatically using the peaks in the low-resolution fluorescence images or manually using the reconstructed super-resolution images. The localization tables were corrected for consecutive localizations before the following analysis. The exact positions of the centers of the clusters were found as the center of mass by the formula

1 $$\vec M = \frac{1}{N}\mathop {\sum }\limits_{i = 1}^N \vec x_i,$$

where *M* is the center of mass of a cluster, *x*_*i*_ is the coordinate of the *i*^th^ localization and *N* is the number of localizations within the clusters. All CENP-A localizations within a circle with a radius of 250 nm around the initial position were taken into account. The distance *r*_*i*_ from *M* to every CENP-A or HJURP localization was then calculated. The histogram of *r*_*i*_ was built from 0 to 200 nm with a bin width of *d* = 20 nm. To obtain a figure proportional to the profile of the rotationally averaged particles, the histogram counts were corrected by dividing them on the surface area *S*_*j*_ of the concentric rings, associated with the histogram bins:

2 $$S_j = \pi (2j - 1)d^2$$

where *j* is the index of the histogram bin (*j* = [1:10]) and d is the bin width. The corrected counts were normalized so that their sum equals unity. The corrected and normalized counts were averaged for all imaged centromeres within a cell. The values of the mean and the standard deviation used for the final graphs were obtained from these averaged profiles, using several different cells at the same time point. The profile of HJURP at 5 h after mitosis (Fig. 4d) was normalized using the normalization coefficient from the HJURP data for 1.5 h after mitosis (Fig. 4c), revealing that at 5 h there is essentially no HJURP at the centromeres.

The calculation of 3D reconstructions based on 2D SMLM data was done using the common line approach (angular reconstitution), a method used in the field of single particle cryo electron microscopy^48^ as implemented in the IMAGIC software^46^ and applied here to SMLM data (to our knowledge one of the first applications together with studies^49,50^ that appeared recently). The fact that the size variations of the clusters are small (see Supplementary Fig. 4 and size analysis above) allowed to perform a 3D reconstruction based on back-projection from 2D images considering that these are 2D projections of the 3D object seen under different angles. The concept of angular reconstitution is that the relative Euler angles between two 2D projections can be determined according to the common line found in their Radon transform (the sinogram). For this, images of the individual CENP-A clusters were handled as single particle images and their relative angles were determined using the angular-reconstitution subroutine of the IMAGIC software. The data set comprises 58 images of different centromeres from one U2OS cell at 1.5 h after mitosis. After assignment of the Euler angles to each individual image, a 3D reconstruction was obtained by weighted, filtered back-projection using the true-threed command. Validation was done by calculating re-projections of the obtained 3D reconstruction according to the same Euler angles, showing a good correlation between input images and corresponding re-projections (Supplementary Fig. 6; see also the Euler angle plot, which shows a good distribution of orientations). The fact that a 3D reconstruction can be obtained from physically different CENP-A clusters in a given cell (i.e., with all centromeres in a similar state) shows that the different centromeric regions in a cell share a common structure, which resembles a globular rosette-like structure that extends to the macroscopic scale considering that the complex reaches dimensions of 300 nm. The 3D reconstruction obtained is consistent with the overall shape of the CENP-A clusters seen for the 2D and 3D SMLM data (Supplementary Fig. 6, Figs. 2 and 3, respectively). This analysis suggests that a common sub-structure exists in all the CENP-A clusters, which is confirmed also by direct 3D SMLM analysis (Fig. 3). The fact that a 3D reconstruction can be obtained from a common line approach also underlines the fact that the objects are structurally similar (at least to a given resolution level, not taking into account a possible structural heterogeneity of finer details which would be averaged out during the 3D reconstruction process). The description of this particular structure applies to early G1 where a defined structural state is observed.

### Reporting summary

Further information on research design is available in the Nature Research Reporting Summary linked to this article.

## Supplementary information

Supplementary Information

Peer Review

Reporting Summary

Source Data

## Data Availability

The datasets generated and analyzed during the current study are available from the corresponding authors on reasonable request. The source data underlying Figs. 2c, 4c and 4d are provided as a Source Data file.
