# Supplementary material for: CENP-A nucleosome clusters form rosette-like structures around HJURP during G1
Source: Nat Commun. 2019 Sep 30;10:4436. doi: 10.1038/s41467-019-12383-3 (PMC6769019; doi:10.1038/s41467-019-12383-3)
Supplement: Supplementary file 1 — Supplementary Information [file 41467_2019_12383_MOESM1_ESM.pdf]

Supplementary Information to

CENP-A nucleosome clusters form rosette-like structures around HJURP during G1

Andronov, Ouararhni *et al.*

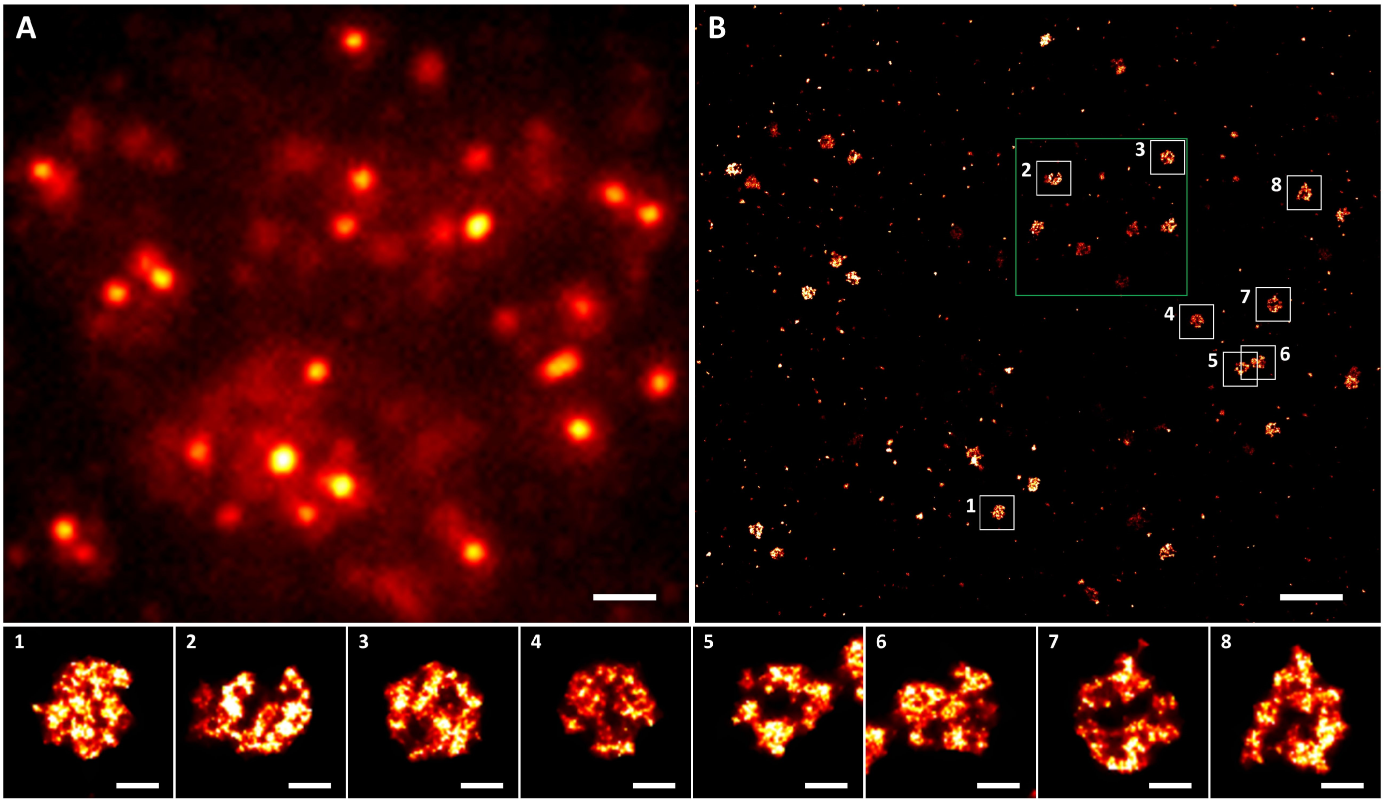

Supplementary Figure 1. SMLM images of centromeres in a non-synchronized HeLa cell. (**A**) Conventional epifluorescence image of CENP-A. (**B**) SMLM image of the same region. Bottom panels **1-8**: zoomed view of several centromeres from this cell. A significant portion of the non-synchronized cells have centromeres of this rosette-like shape, suggesting that they are formed in a particular phase of the cell cycle. The green rectangle represents a region that is zoomed in **Supplementary Figure 2**. Scale bars, 500 nm (A-B) and 100 nm (1-8).

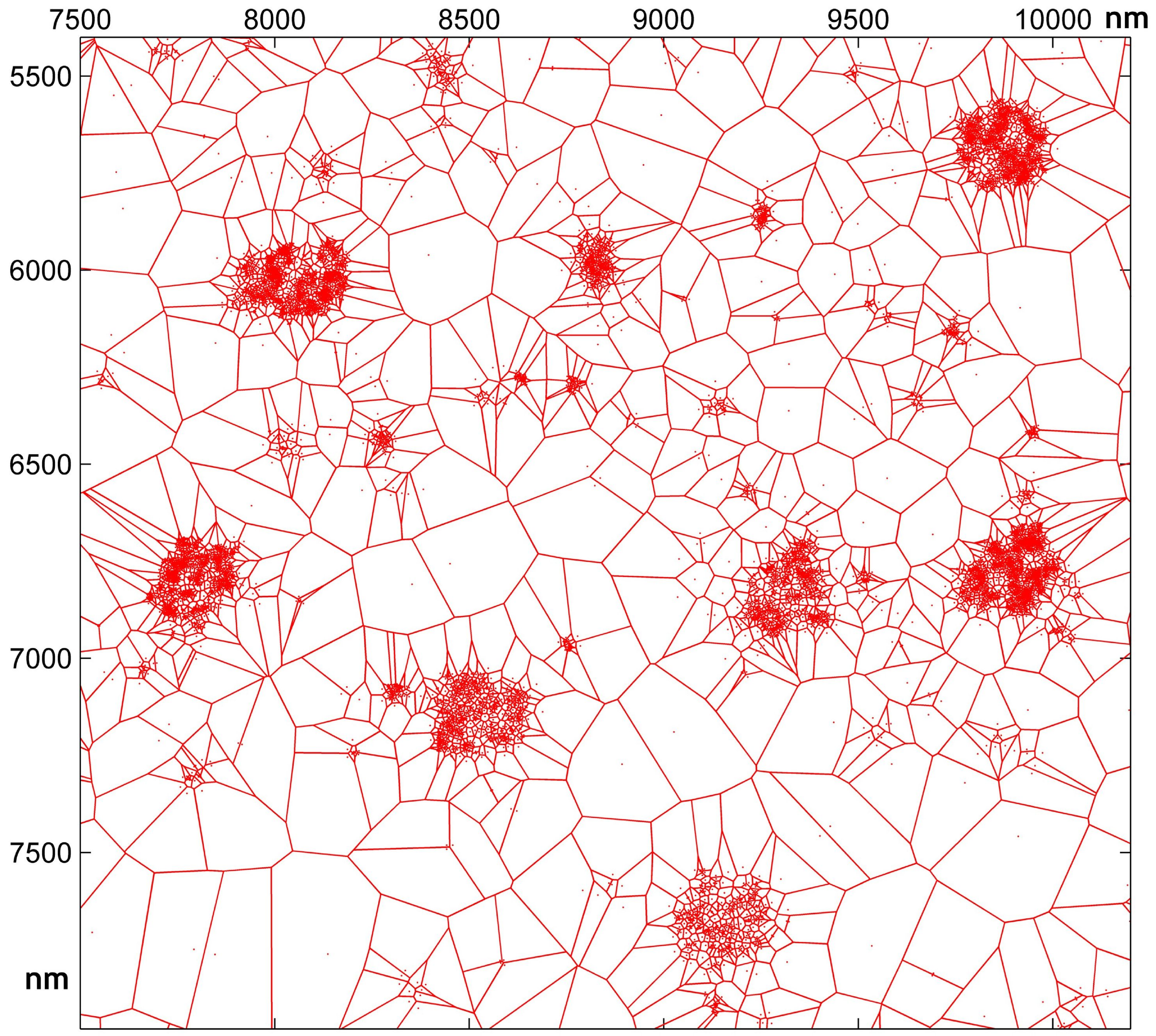

Supplementary Figure 2. A Voronoi diagram built on the SMLM data in **Supplementary Figure 1**, revealing the presence of intense CENP-A clusters with several hundreds of localizations inside.

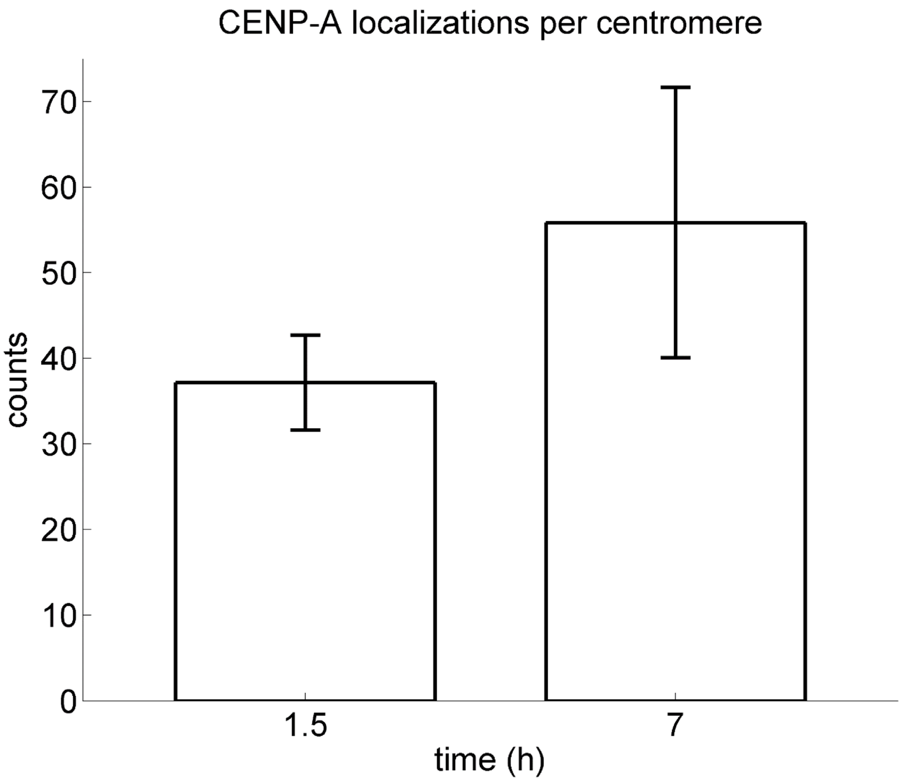

Supplementary Figure 3. Localizations of CENP-A per centromere at 1.5 and 7 hours after mitosis, using a stable sell line expressing mEOS2-CENP-A. A 1.5 times increase due to the deposition of new CENP-A can be seen. The increase is less than two-fold, because some CENP-A might have been already deposited before the 1.5 hours' time point (experimentally after cell adhesion, see methods). P-value, 0.0024, two-tailed Student's t-test. 10 cells were used for each time point.

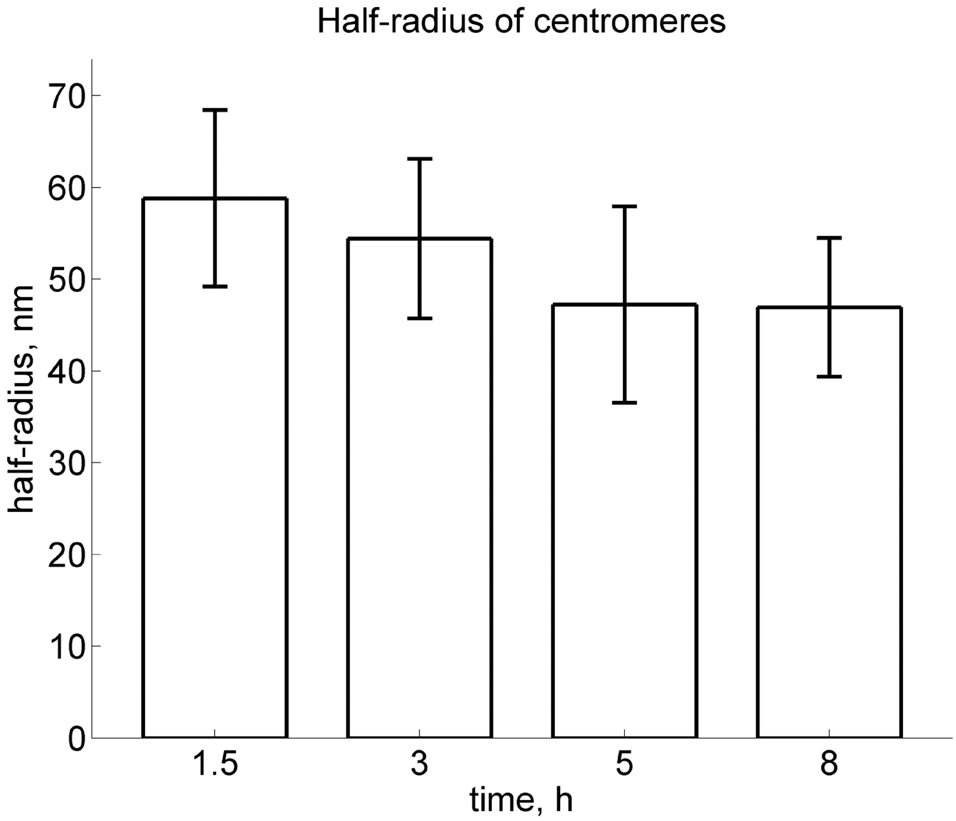

Supplementary Figure 4. Half-radius of centromeres at different time points, measured as the radius where the cumulative profile of the rotationally averaged images of centromeres reaches 50% (based on data presented in **Fig. 2**). A decrease in radius can be seen, indicating a global compaction of centromeres. Error bars represent the standard deviation between individual centromere sizes (taking into account all centromeres from several cells synchronized at a given time point).

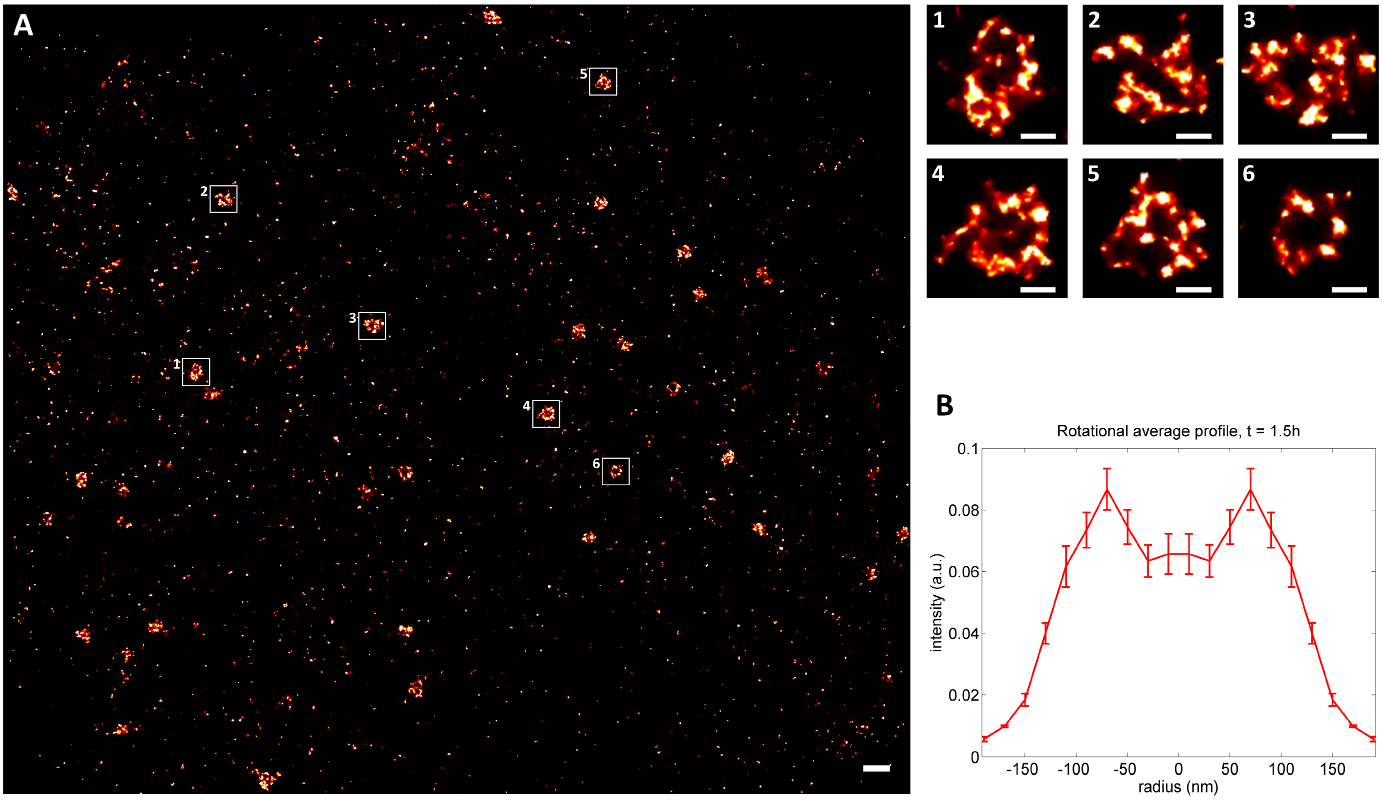

Supplementary Figure 5. SMLM (PALM) imaging of CENP-A, labelled with the mEOS2 photo-convertible protein at 1.5 hours after mitosis using U2OS cells stably expressing mEOS2-CENP-A. (**A**) A super-resolution image of two adjacent cells. (1-6) Zoomed-in centromeres. (**B**) Rotationally averaged profile of centromere images from 4 different cells at 1.5 hours after mitosis. Scale bars, 500 nm (A) and 100 nm (1-6).

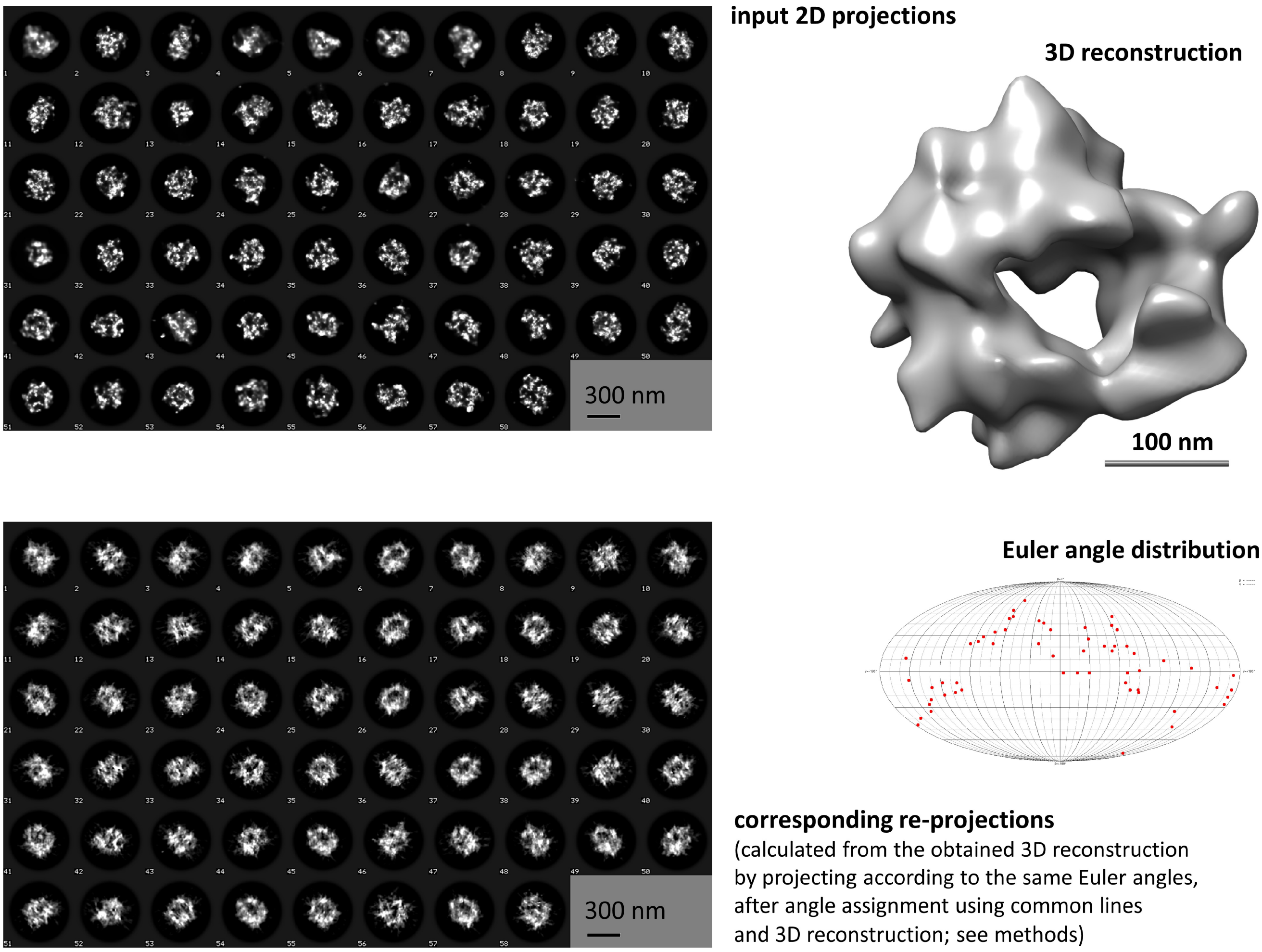

Supplementary Figure 6. 3D reconstruction of CENP-A clusters using the common line approach, a technique used in single particle cryo electron microscopy to assign relative angles of 2D projection images and obtain a 3D map (*48*) as implemented in the IMAGIC software (*46*). (top) Images of different centromeres from one U2OS cell at 1.5 hours after mitosis; individual centromere images were handled as single particle images for the angle assignment. (right) 3D map reconstructed from these centromere images obtained by back-projection of the input images into a 3D reconstruction (*46*); the reconstruction shows a relatively globular, spherical shape with a cavity in the center which resembles a rosette; the Euler plot below (X_euler software, Orlov & Klaholz, unpublished) shows a good angular distribution without preferential orientations (which could create distortions), consistent with the observation of a globular shape rather than an elongated (distorted) barrel-like object; the absence of preferred orientations also indicates that imaging was not a limiting factor in terms of, for example, effective axial localization range or potential poorer axial resolution, also considering that these effects would be averaged out in the reconstruction due to the fact that the individual objects have different orientations in the sample when being imaged. (bottom) Corresponding re-projections of the 3D reconstruction in the directions of the Euler angles of the input particles, to be compared with the input images at the top (see methods), which shows the correctness of the Euler angle assignment and validates the consistency between input projections and the 3D reconstruction.

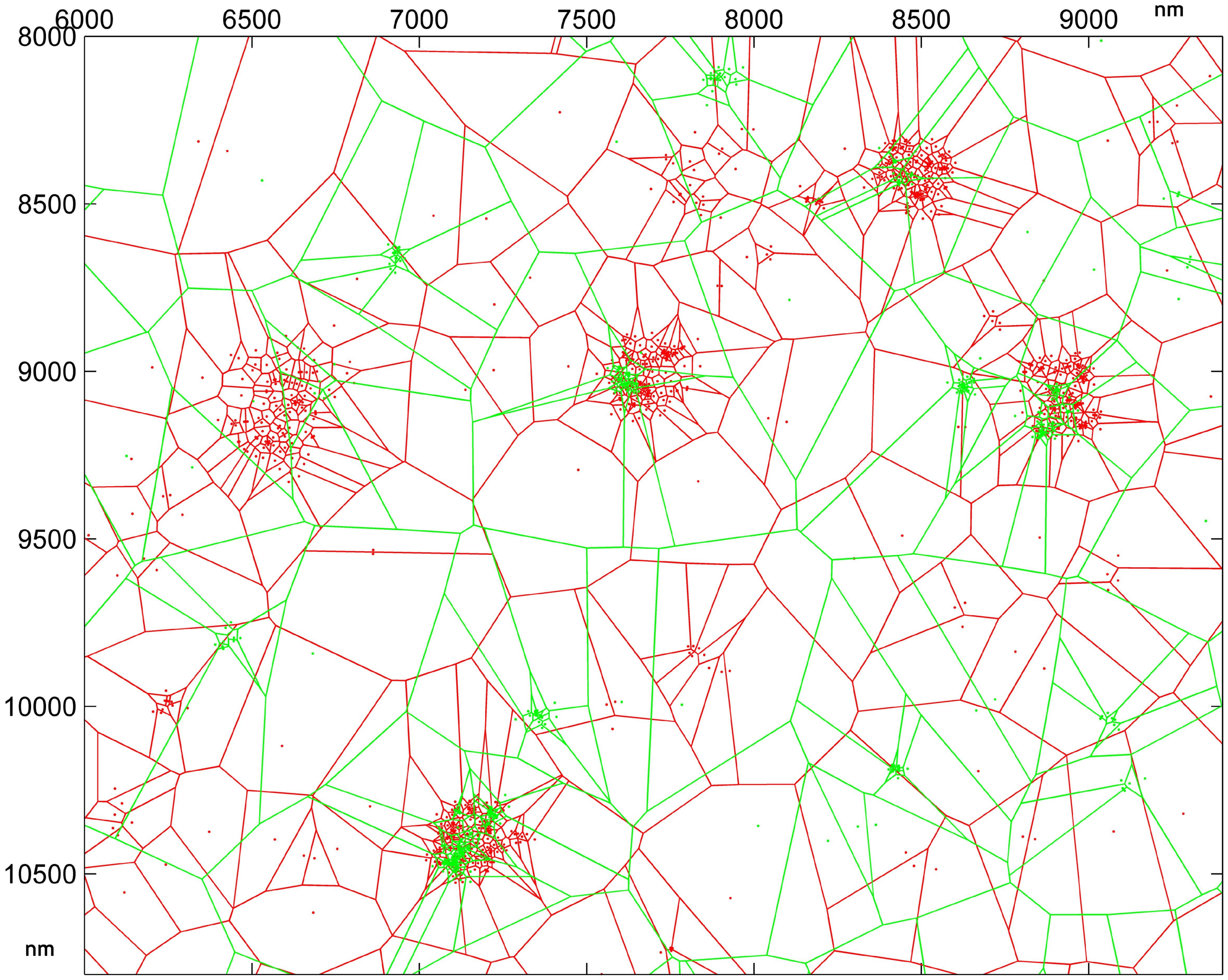

Supplementary Figure 7. Voronoi diagrams built on SMLM data from **Fig. 4**. CENP-A labelling is shown in red and HJURP labelling is shown in green.

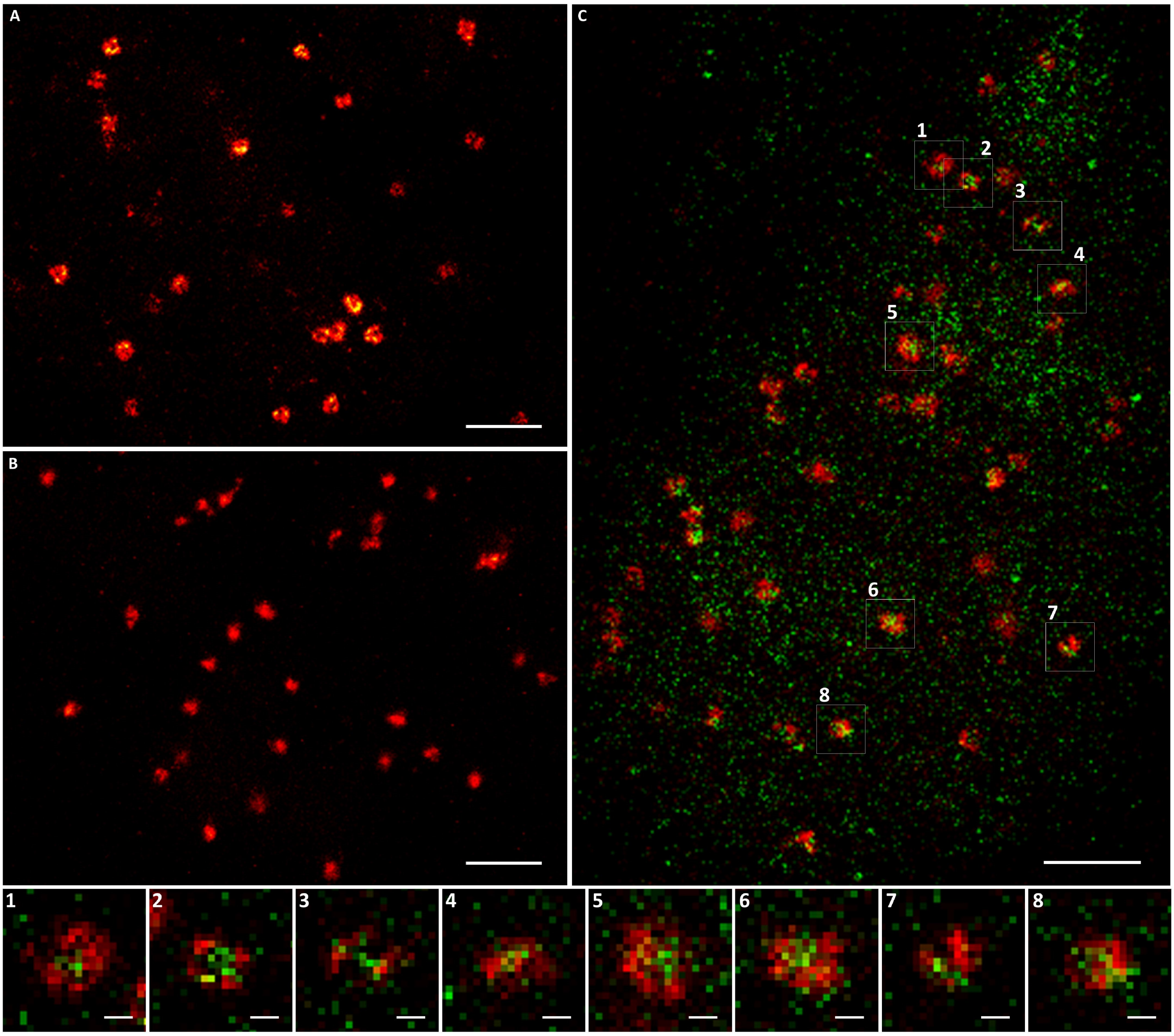

Supplementary Figure 8. STED imaging of CENP-A and HJURP proteins (synchronized U2OS cells). (**A**) CENP-A forms hollow clusters in a cell fixed at 1.5 hours after mitosis. (**B**) At 8 hours after mitosis clusters become less sharp without the central hole. (**C**) HJURP (green) forms smaller clusters that are often situated in the middle of the CENP-A clusters (red). (Bottom panels **1**–**8**) Zoomed-in centromeres from panel C. Scale bars, 1 μm (A-C) and 100 nm (bottom panels 1–8).

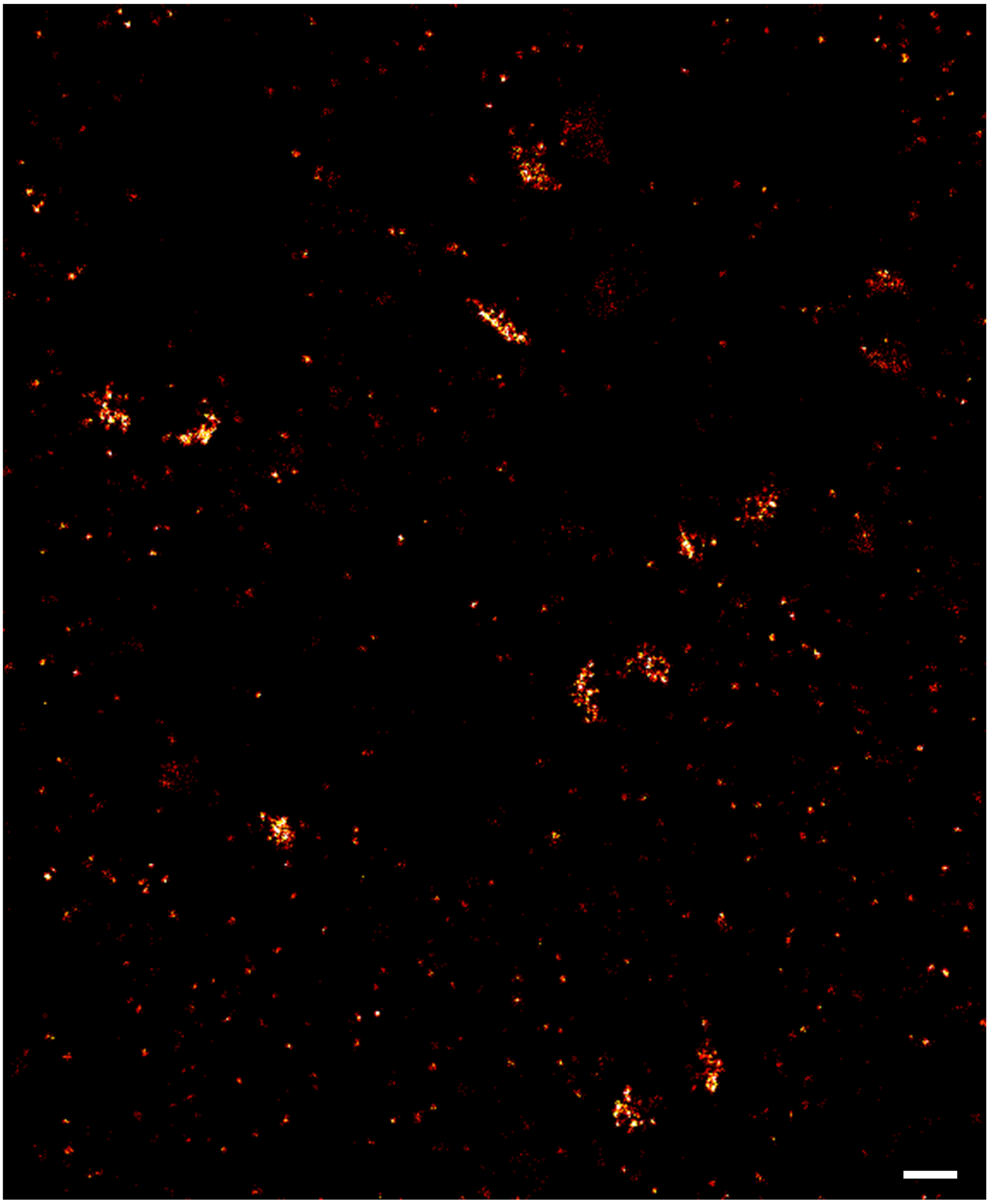

Supplementary Figure 9. SMLM imaging of CENP-A in a mitotic U2OS cell (prometaphase). Elongated and round structures can be seen, which is compatible with the disk-like organization of CENP-A during mitosis[24-25]. CENP-A was labelled with Alexa-647-conjugated secondary antibodies. Scale bar, 500 nm.

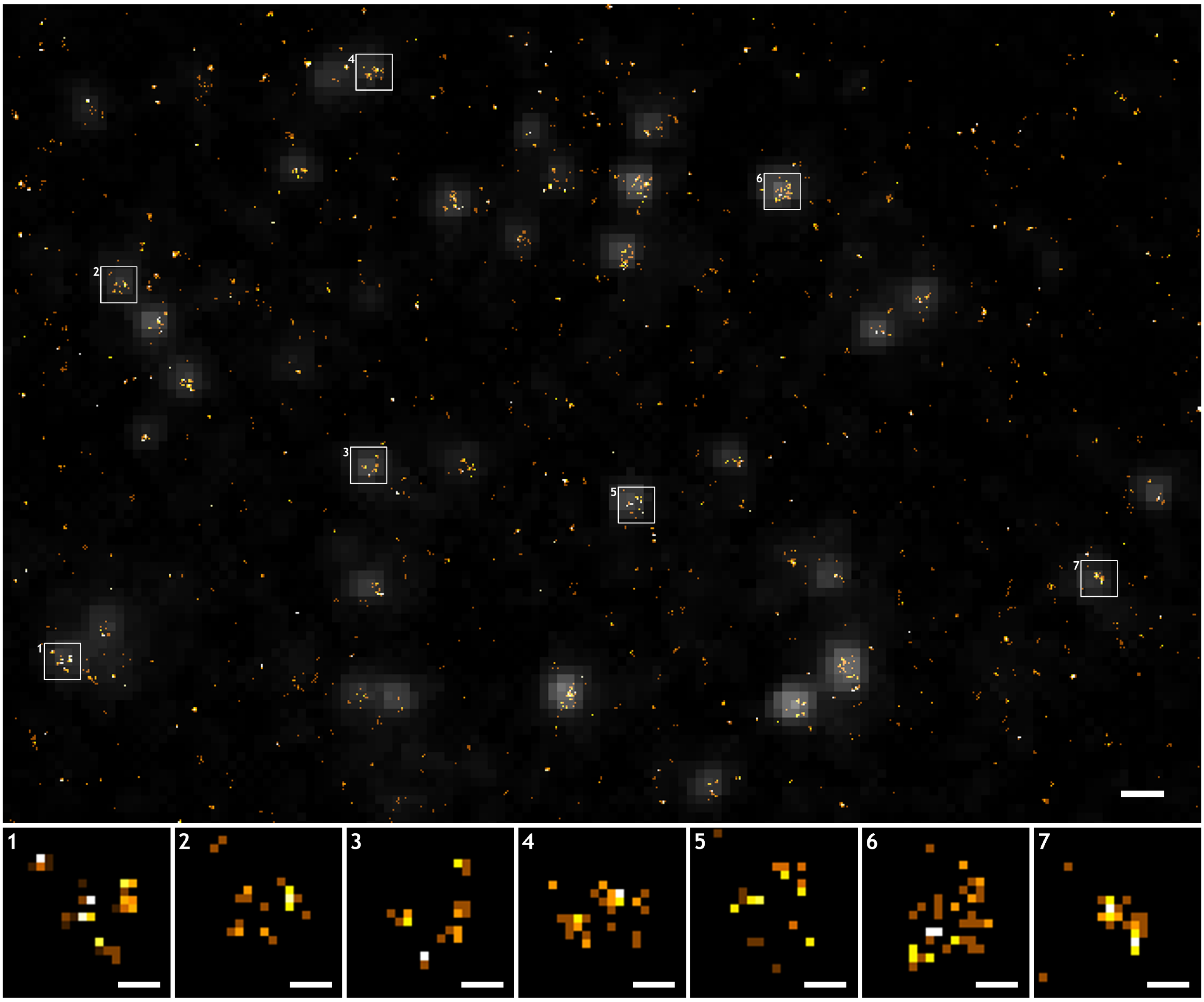

Supplementary Figure 10. SMLM imaging of CENP-A, labelled with the mEOS2 photo-convertible protein at 7 hours after mitosis using U2OS cells, transiently transfected with mEOS2-CENP-A in the S phase of the previous cell cycle. (Top) A super-resolution image of one cell nucleus in yellow with conventional fluorescence microscopy image overlaid in gray. (Bottom panels 1-7) Zoomed-in centromeres. Scale bars, 500 nm (top) and 100 nm (bottom panels 1-7).

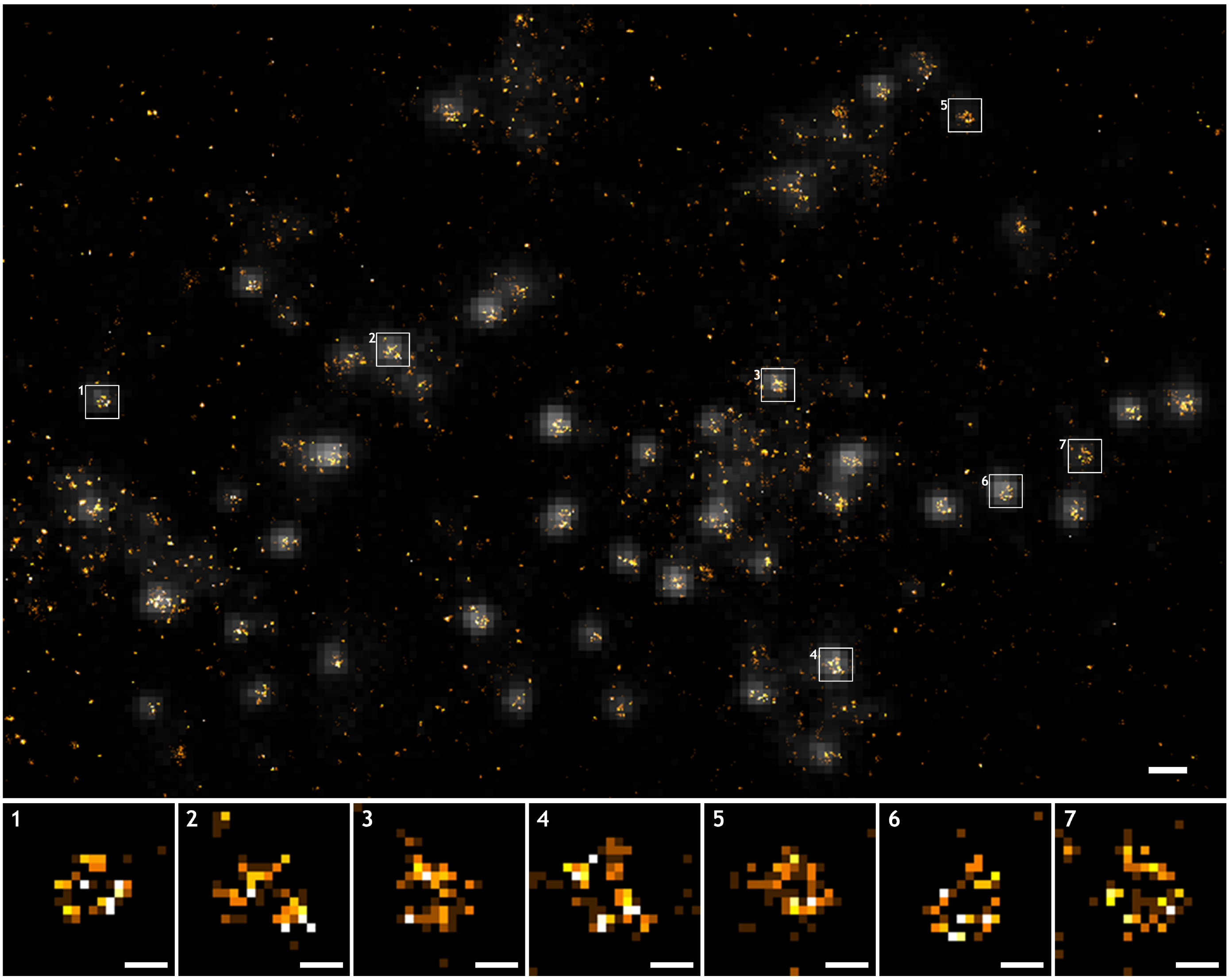

Supplementary Figure 11. SMLM imaging of CENP-A in a mouse embryonic fibroblast (MEF) cell, stably expressing CENPA-HA tag, and fixed 1 hour after mitosis. The HA tag (CENP-A) was detected with Alexa-647-conjugated secondary antibodies. (Top) A super-resolution image of one cell nucleus in yellow with conventional fluorescence microscopy image overlaid in gray. (Bottom panels 1-7) Zoomed-in centromeres. Scale bars, 500 nm (top) and 100 nm (bottom panels 1-7).

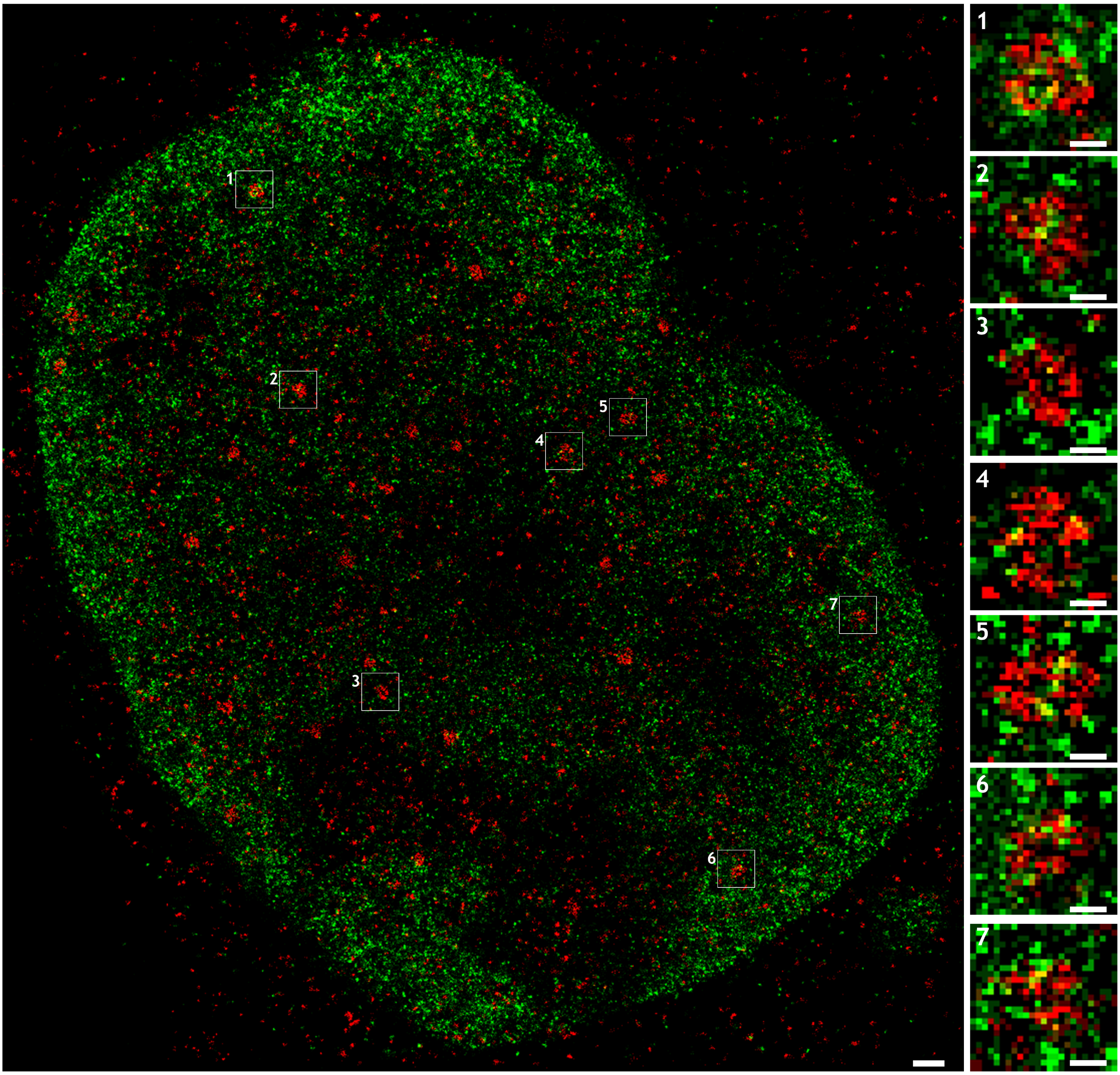

Supplementary Figure 12. SMLM imaging of CENP-A (red) with H3.3 (green) in a U2OS cell, stably expressing H3.3-HA tag. The HA tag was detected with Alexa-488-conjugated secondary antibodies and CENP-A was detected with Alexa-647-conjugated secondary antibodies. Scale bars, 500 nm (left) and 100 nm (right panels 1-7).

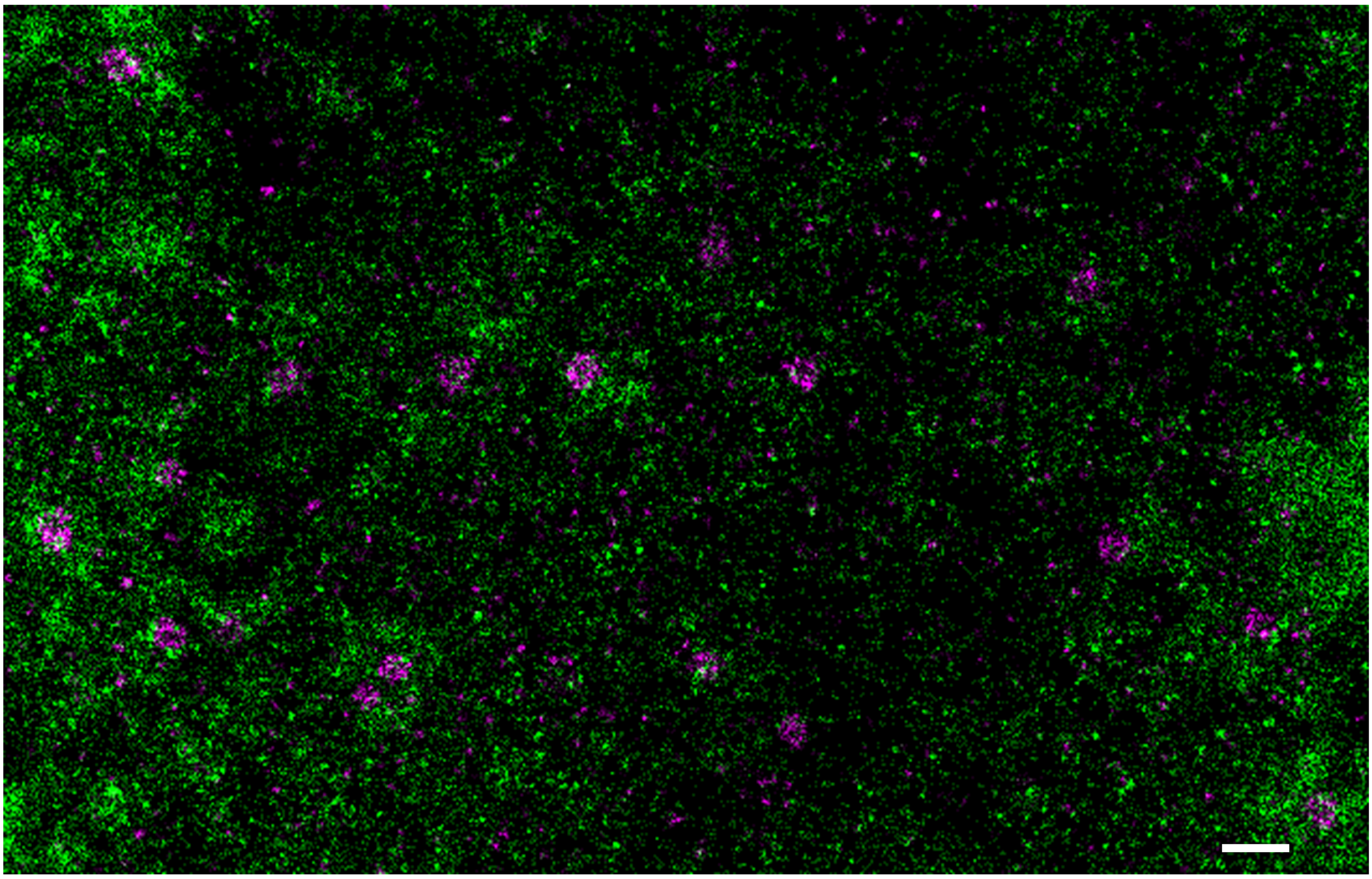

Supplementary Figure 13. SMLM imaging of CENP-A (violet) with DNA (green) in a U2OS cell at 1.5 hours after mitosis. DNA staining is diffused in the nucleus, as expected for interphase. CENP-A was detected with Alexa-647-conjugated secondary antibodies and DNA was stained with YoYo-1. Scale bar, 500 nm.

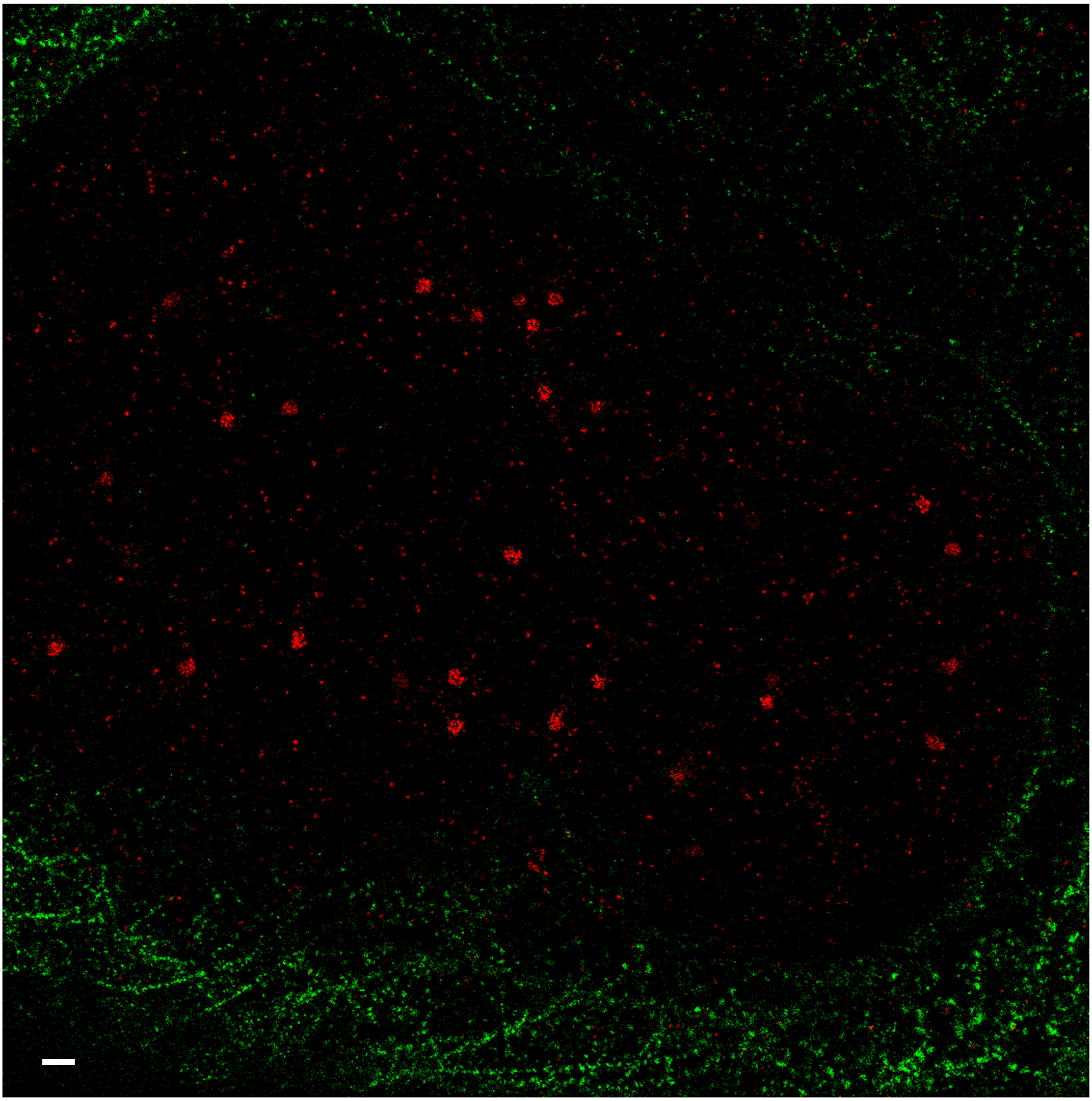

Supplementary Figure 14. SMLM imaging of CENP-A (red) with β-tubulin (green) in a U2OS cell at 1.5 hours after mitosis. Microtubules are absent in the cell nucleus, as expected for interphase. CENP-A was detected with Alexa-647-conjugated secondary antibodies and β-tubulin was detected with Alexa-488-conjugated secondary antibodies. Scale bar, 500 nm.

Antibody	Producer	Reference
Rabbit anti-CENP-A	Sigma-Aldrich	07-574
Rat anti-HA high affinity clone 3F10	Sigma-Aldrich	11867423001
Mouse anti-β-tubulin	IGBMC	1TUB-2A2-4-3
Mouse anti-HJURP	EPIGEX	EPX-H80
Alexa Fluor 647 goat anti-Mouse IgG1	Fisher Scientific	A-21240
Alexa Fluor 647 chicken anti-Rat IgG	Fisher Scientific	A-21472
Alexa Fluor 647 goat anti-Rabbit IgG	Fisher Scientific	A-21245
Alexa Fluor 555 goat anti-Mouse IgG (H+L)	Fisher Scientific	A-21424
Alexa Fluor 555 goat anti-Rabbit IgG (H+L)	Fisher Scientific	A-21428
Alexa Fluor 488 goat anti-Mouse IgG (H+L)	Fisher Scientific	A-11001
Alexa Fluor 488 goat anti-Rabbit IgG (H+L)	Fisher Scientific	A-11008

Supplementary Table 1. Antibodies used in this study.

Target – fluorophore	Imaging buffer	Experiments	Cells	Localizations per cell	Median photon count	$FRC_{1/7}$ resolution, nm
CENP-A – Alexa Fluor 647	Vectashield/TDE	7	421	30 000	2 000	43
	OxEA		8	40 000	1 000	52
	MEA		58	5 000	550	70
CENP-A – Alexa Fluor 555	Vectashield/TDE	3	55	10 000	1 200	60
HJURP – Alexa Fluor 488	MEA	2	59	5 000	400	50
HJURP – Alexa Fluor 647	Vectashield/TDE	2	43	10 000	1 700	40
CENP-A – mEOS2	PBS	3	34	5 000	1 500	90
	Vectashield/TDE		9	15 000	2 500	40
β-tubulin – Alexa Fluor 488	OxEA	1	4	100 000	1 500	60
DNA – YoYo-1	OxEA	1	3	100 000	1 200	120
H3.3-HA – Alexa Fluor 488	OxEA	1	4	250 000	1 200	65

Supplementary Table 2. Quality parameters of the SMLM data used in this study. “Experiments” denote the number of biologically independent experiments and “Cells” denote the total number of imaged cells. Typical values are shown in columns 5-7.
