# Supplementary material for: CENP-A nucleosome clusters form rosette-like structures around HJURP during G1
Source: Nat Commun. 2019 Sep 30;10:4436. doi: 10.1038/s41467-019-12383-3 (PMC6769019; doi:10.1038/s41467-019-12383-3)
Supplement: Supplementary file 3 — Reporting Summary [file 41467_2019_12383_MOESM3_ESM.pdf]

nature research

Corresponding author(s): Bruno Klaholz and Ali Hamiche

Last updated by author(s): Apr 9, 2019

Reporting Summary

Nature Research wishes to improve the reproducibility of the work that we publish. This form provides structure for consistency and transparency in reporting. For further information on Nature Research policies, see Authors & Referees and the Editorial Policy Checklist .

Statistics

For all statistical analyses, confirm that the following items are present in the figure legend, table legend, main text, or Methods section.

n/a	Confirmed	
☐	☒	The exact sample size (n) for each experimental group/condition, given as a discrete number and unit of measurement
☐	☒	A statement on whether measurements were taken from distinct samples or whether the same sample was measured repeatedly
☐	☒	The statistical test(s) used AND whether they are one- or two-sided<br>*Only common tests should be described solely by name; describe more complex techniques in the Methods section.*
☒	☐	A description of all covariates tested
☐	☒	A description of any assumptions or corrections, such as tests of normality and adjustment for multiple comparisons
☐	☒	A full description of the statistical parameters including central tendency (e.g. means) or other basic estimates (e.g. regression coefficient) AND variation (e.g. standard deviation) or associated estimates of uncertainty (e.g. confidence intervals)
☐	☒	For null hypothesis testing, the test statistic (e.g. F, t, r) with confidence intervals, effect sizes, degrees of freedom and P value noted<br>*Give P values as exact values whenever suitable.*
☒	☐	For Bayesian analysis, information on the choice of priors and Markov chain Monte Carlo settings
☒	☐	For hierarchical and complex designs, identification of the appropriate level for tests and full reporting of outcomes
☐	☒	Estimates of effect sizes (e.g. Cohen's d, Pearson's r), indicating how they were calculated

Our web collection on statistics for biologists contains articles on many of the points above.

Software and code

Policy information about availability of computer code

Data collection	Leica Application Suite X 1.9.0.13747
Data analysis	SharpViSu 1.3

For manuscripts utilizing custom algorithms or software that are central to the research but not yet described in published literature, software must be made available to editors/reviewers. We strongly encourage code deposition in a community repository (e.g. GitHub). See the Nature Research guidelines for submitting code & software for further information.

Data

Policy information about availability of data

All manuscripts must include a data availability statement. This statement should provide the following information, where applicable:

- Accession codes, unique identifiers, or web links for publicly available datasets
- A list of figures that have associated raw data
- A description of any restrictions on data availability

Provide your data availability statement here.

Field-specific reporting

Please select the one below that is the best fit for your research. If you are not sure, read the appropriate sections before making your selection.

☒ Life sciences ☐ Behavioural & social sciences ☐ Ecological, evolutionary & environmental sciences

For a reference copy of the document with all sections, see nature.com/documents/nr-reporting-summary-flat.pdf

Life sciences study design

All studies must disclose on these points even when the disclosure is negative.

Sample size	*Describe how sample size was determined, detailing any statistical methods used to predetermine sample size OR if no sample-size calculation was performed, describe how sample sizes were chosen and provide a rationale for why these sample sizes are sufficient.*
Data exclusions	*Describe any data exclusions. If no data were excluded from the analyses, state so OR if data were excluded, describe the exclusions and the rationale behind them, indicating whether exclusion criteria were pre-established.*
Replication	*Describe the measures taken to verify the reproducibility of the experimental findings. If all attempts at replication were successful, confirm this OR if there are any findings that were not replicated or cannot be reproduced, note this and describe why.*
Randomization	*Describe how samples/organisms/participants were allocated into experimental groups. If allocation was not random, describe how covariates were controlled OR if this is not relevant to your study, explain why.*
Blinding	*Describe whether the investigators were blinded to group allocation during data collection and/or analysis. If blinding was not possible, describe why OR explain why blinding was not relevant to your study.*

Reporting for specific materials, systems and methods

We require information from authors about some types of materials, experimental systems and methods used in many studies. Here, indicate whether each material, system or method listed is relevant to your study. If you are not sure if a list item applies to your research, read the appropriate section before selecting a response.

Materials & experimental systems

n/a	Involved in the study	
☐	☒	Antibodies
☐	☒	Eukaryotic cell lines
☒	☐	Palaeontology
☒	☐	Animals and other organisms
☒	☐	Human research participants
☒	☐	Clinical data

Methods

n/a	Involved in the study	
☒	☐	ChIP-seq
☒	☐	Flow cytometry
☒	☐	MRI-based neuroimaging

Antibodies

Antibodies used	Rabbit anti-CENP-A # 07-574 (Sigma-Aldrich), Rat anti-HA high affinity clone 3F10 #11867423001 (Sigma-Aldrich), Mouse anti-β-tubulin #1TUB-2A2-4-3 (IGBMC), Mouse anti-HJURP #EPX-H80 (EPIGEX), Alexa Fluor 647 goat anti-Mouse IgG1 # A-21240 (Fisher Scientific), Alexa Fluor 647 chicken anti-Rat IgG # A-21472 (Fisher Scientific), Alexa Fluor 647 goat anti-Rabbit IgG # A-21245 (Fisher Scientific), Alexa Fluor 555 goat anti-Mouse IgG (H+L) # A-21424 (Fisher Scientific), Alexa Fluor 555 goat anti-Rabbit IgG (H+L) # A-21428 (Fisher Scientific), Alexa Fluor 488 goat anti-Mouse IgG (H+L) # A-11001 (Fisher Scientific), Alexa Fluor 488 goat anti-Rabbit IgG (H+L) # A-11008 (Fisher Scientific),
Validation	All the antibodies were validated by the manufacturers by western-blotting and imaging

Eukaryotic cell lines

Policy information about cell lines

Cell line source(s)	HeLa and U2OS from ATCC. MEF cell lines derived from C57BL/6 mice.
Authentication	HeLa S3 (ATCC® CCL-2.2™), U-2 OS (ATCC HTB-96), MEF cell lines derived from day 13 mouse embryos C57BL/6 m
Mycoplasma contamination	negative for mycoplasma contaminations
Commonly misidentified lines (See ICLAC register)	n/a
