# Supplementary material for: CENP-A nucleosome clusters form rosette-like structures around HJURP during G1
Source: Nat Commun. 2019 Sep 30;10:4436. doi: 10.1038/s41467-019-12383-3 (PMC6769019; doi:10.1038/s41467-019-12383-3)
Supplement: Supplementary file 2 — Peer Review [file 41467_2019_12383_MOESM2_ESM.pdf]

Reviewers' comments:

Reviewer #1 (Remarks to the Author):

Andronov et al visualize centromeric histone CENP-A and its chaperone HJURP using single-molecule localization microscopy (SMLM). In various organisms, CENP-A on each chromosome has been seen only as a round spot due to the diffraction limit resolution of conventional immunofluorescence microscopy. Using the higher resolution power stochastic optical reconstruction microscopy (STORM), Andronov et al were able to further zoom in into the centromere of a single chromosome. They observe that during early G1, CENP-A forms ring-like rosette structure at each centromere and HJURP occupies the center of this ring. Authors propose to call this structure “assemblosome”. This ring-like rosette structure is converted into a spherical structure when cells transition from early G1 into late G1 stage. Moreover, the authors suggest that the HJURP cluster in the cavity of the ring might serve as a nucleation point for CENP-A deposition. The observation advances the understanding of how CENP-A is arranged at the centromeres. However, further investigation of the ring structure is needed to place their observation in the functional context and to create a complete story for publication.
Specific concerns:
1. How is this rosette shaped structure placed on the sister chromatids and how does it interact with microtubules? STORM co-localization of CENP-A with DNA and microtubule staining should answer this question without any difficulty.
2. During ring (early G1) to sphere (late G1) transition, does the central cavity fill with additionally deposited CENP-A or does the sphere result simply from the re-organization of the ring? How does the diameter of the ring changes from early G1 to late G1?
3. Understanding the spatial relationship between CENP-A and H3 chromatin has been of high interest to the centromeric field. However, lack of tools has hindered gaining insights into this aspect. STORM co-localization of CENP-A and H3 should reveal how H3 is arranged both linearly and in 3D at centromeric regions. If not the exact placement of H3 alongside CENP-A, authors should at least find out if H3 is present on this ring or not. Or it may reveal if the H3 coats inside or outside of the ring.
4. Figure 3B – I don't understand the point of showing all the panels. They all look very similar to me. Please explain the differences if any. Figure 3C - A schematic inset for each panel would be helpful to show the progression of the density distribution from a shell to a disc shape.
5. A discussion about the relevance of CENP-A ring in the kinetochore structure and function is completely missing. The authors should discuss what is already known from previous STORM imaging of centromeric proteins. For example, super-resolution images of mitotic centromeres by Wynne and Funabiki (PMID: 27170176) has revealed ring-shaped kinetochores (stained with Ndc80, CENP-E, BubR1) in which inner kinetochore proteins (CENP-A, CENP-C & Mis12) occupy a central position. So the ring shape seems to be a structural feature of the entire kinetochore where the inner kinetochore components including CENP-A might undergo compaction and acquire a spheroid shape. Since CENP-A is the innermost protein in this ring, it would make sense for HJURP to bind and access centromeric chromatin from the inside of the ring. The rosette by itself might not have an exclusive function in CENP-A loading by HJURP so the term assemblosome is premature.
6. For the amount of information available in the current version of the manuscript, it can be significantly shortened. Line 69 – please replace “thundering”.

Reviewer #2 (Remarks to the Author):

Andronov et al. CENP-A nucleosome clusters form rosette-like structures around HJURP during G1.

In this paper the authors present super-resolution imaging data of CENP-A and HJURP in human cells at different cell stages. They show that the structure of the CENP-A clusters changes will cell cycle and that this is corelated to the presence of HJURP at the centres of these clusters.

Overall this is an interesting paper with potential to significant extend our understanding of the biological function of CENP-A, its recruitment to the centromere, and hence its involvement in chromosome segregation. Broadly the paper is extremely well written with clear descriptions and good figures. The statistics, such as N numbers and SEM versus SD are clearly stated.

I have a few minor comments.

1) The authors show a large number of SMLM images but don't give any indications as to the quality of the data fitting. They should state some information about number of localisations and average localisation precision to give an idea about the real data quality. Alternatively, they could use a technique such as Fourier ring correlation to measure actual image resolution.
2) In the final section the authors state "This suggests that the newly discovered specific rosette-like structure of the HJURP- centromeric nucleosome complexes is probably a common feature of eukaryotic cells at the G1 phase of the cell cycle." They have examined this structure in two human derived cell lines. It would be interesting to show that this is common feature across many eukaryotes but this paper does not show this.
3) They suggest in the methods section that this is the first application of single particle reconstruction methods from cryoEM to SMLM data. This is not true, eg see DOI: 10.1109/TMI.2018.2795464

Overall I think this paper is suitable for publication with minor updates.

Reviewer #3 (Remarks to the Author):

The authors present an interesting study on the distribution of CENP-A in G1. In general the work is good, but not as thorough as I would expect for Nature Communications. There are some reasonably major issues that should be addressed prior to acceptance for publication. There are also a number of spelling and idiomatic inconsistencies (a few of which I've listed at the end of this review) and I'd encourage the authors to thoroughly proof-read their work before submitting a revised version. Starting with my scientific concerns.

- When imaging shortly after mitosis it is reasonable to assume that much of the kinetochore is still be intact. From previous EM studies this has been shown to be an incredibly densely packed structure, and one might reasonably expect access by antibodies to be impaired within the centre of the kinetochore. A very plausible explanation of the observations presented here would be if access to the centrally located CENP-A only became possible as the kinetochore disassembles throughout G1. The authors should eliminate this as a possible cause (or at the very least discuss it). The most compelling test for this would be to use a tagged version of CENP-A (e.g. with a SNAP or HALO tag – both of which would allow labelling with STORM compatible dyes, or with a switchable fluorescent protein).

- Previous (mainly EM, but also confocal measurements based on the fact that CENP-A typically presents as a spot which is a little bit larger than the diffraction limit) work has established that the centromeric regions have a size of ~200-500nm, and that there is a lot of variation between chromosomes within one cell. The amount of variation that is known to exist in the structure makes any class averaging efforts extremely tenuous. As it stands there is very little justification for using the alignment and back projection methods used to obtain the data in Sup. Fig 3, which are based on the assumption that each of the structures being averaged is essentially identical. Critically the conclusions do not depend on this data – the 2D and 3D dSTORM images provide reasonably compelling evidence for reduced labelling towards the centre of the centromeres. Rather I feel that it's inclusion reduces my confidence in the rest of the work (if the authors are happy to apply an analysis method for which the fundamental assumptions are not met, can I have any confidence in the rest of their data?).

- My class averaging concerns also apply (to a considerably lesser extent) to the rotationally averaged

data in Fig 3. If object size varies, these become an imperfect tool. In this case, however, it is probably good enough. That said a quantification of the distribution of CENP-A cluster sizes at each time point would be a useful control. A broadening in the distribution of radii of an annular structure would also lead to a filling in of the centre of a rotational average and this quantification should be able to put a limit on the magnitude of such an effect.

- The authors assert that HJURP localizes to the centre of the CENP-A clusters, but do not present any quantitative evidence to support this (Fig 4 is suggestive, but not at all quantitative). A simple solution would be to also calculate the rotational average of HJURP (using the centres inferred for CENP-A) and to plot both radial profiles in the same graph.

- Previous studies have suggested that the kinetochore (and by extension the centromeric chromatin) are a flat, disc-like structure. It would be interesting to discuss the results in this context, and more generally in the context of previous EM studies. How much of the axial extent of the stuctures in Fig 2 is due to the axial localization precision and/or systematic errors?

- The authors talk about numbers of CENP-A (L 144) specifying that the clusters contained several hundred CENP-A, but do not detail how they got these numbers. Counting molecules in dSTORM (and other localization microscopies) is not simple, particularly when combined with antibody labelling, and any attempt to count molecules need to be described in detail. The authors should either describe in detail how they arrived at the count, complete with the relevant calibration measurements, or remove this detail from the paper (as the conclusions do not depend on it).

- A useful addition would be a characterization of the relative amounts of CENP-A in a cluster at each time point (this is much easier than absolute molecule counts). The authors stress the fact that newly synthesized CENP-A is being incorporated and a quantification of the relative amounts would help this story (you would expect roughly 2x as many localizations from clusters towards the end of the imaging period, whereas a naïve examination of the example structures in Fig 3A would suggest that this more than doubles – potentially supporting for the accessibility argument.).

Minor points

Fig 3: consider using an alternative colour map (e.g. greyscale) for the rotational averages. The "jet" color map is a poor choice for representing changes in density, as the brightest area appears darker than much of the rest of the images.

L 69 – "thundering importance" – not idiomatic
L 79 – "down to" implies a lower bound, not a range
L 88 – "peculiar"- this implies abnormal – "special" might be more idiomatic
L 93 – spelling – "Alexa Fluor"

L 98 – "using adaptive optics" – clarified in methods, but unclear what adaptive optics is used for (generating the astigmatism) at this point. Would be clearer if omitted here and just left in methods.

L148/149 – "colocalization analysis" – This would usually imply some form of quantification of the spatial relationship between the two labels. You just present two colour images, and don't present the results of any colocalization analysis.

L 367 – "exposure" time (not exposition)

L 389 – events
L 391 – single molecule return (not returns)

L407 – molecule chaining – also need to specify time window (i.e. how many frames can a molecule be dark and still be linked and averaged)?

Response to the comments of the reviewers

Reviewer #1 (Remarks to the Author):

Andronov et al visualize centromeric histone CENP-A and its chaperone HJURP using single-molecule localization microscopy (SMLM). In various organisms, CENP-A on each chromosome has been seen only as a round spot due to the diffraction limit resolution of conventional immunofluorescence microscopy. Using the higher resolution power stochastic optical reconstruction microscopy (STORM), Andronov et al were able to further zoom in into the centromere of a single chromosome. They observe that during early G1, CENP-A forms ring-like rosette structure at each centromere and HJURP occupies the center of this ring. Authors propose to call this structure "assemblosome". This ring-like rosette structure is converted into a spherical structure when cells transition from early G1 into late G1 stage. Moreover, the authors suggest that the HJURP cluster in the cavity of the ring might serve as a nucleation point for CENP-A deposition. The observation advances the understanding of how CENP-A is arranged at the centromeres. However, further investigation of the ring structure is needed to place their observation in the functional context and to create a complete story for publication.

A> We thank the referee for the comments. We have now done a whole series of new SMLM experiments, including with stable cell lines, to address these points, as detailed below. These include microtubule, H3.3 and DNA labeling, and CENP-A labeling during mitosis.

Specific concerns:
1. How is this rosette shaped structure placed on the sister chromatids and how does it interact with microtubules? STORM co-localization of CENP-A with DNA and microtubule staining should answer this question without any difficulty.

A> Because the analysis focuses on the structure of the centromere chromatin in G1 phase (interphase), there should be no sister chromatids nor microtubules inside the cell nucleus. We nevertheless imaged CENP-A with microtubules and with DNA. As expected, the images show that there is indeed no microtubules within the cell nucleus in early G1 (see new Suppl. Fig. S12); regarding DNA, the staining is diffused within the nucleus, as expected, and does not allow to find any peculiarities (see new Suppl. Fig. S11). We also did the experiment to localize CENP-A in mitosis (where sister chromatids can be seen indeed, see new Suppl. Fig. S9). Interestingly, mitotic centromeres adopt a rod-like structure compatible with what is known for the centromeric

organization during mitosis (see new Suppl. Fig. S9). These additional data underline and confirm the description of the CENP-A cluster during early G1.

2. During ring (early G1) to sphere (late G1) transition, does the central cavity fill with additionally deposited CENP-A or does the sphere result simply from the re-organization of the ring?

A> To answer this question, we transiently transfected cells synchronized in S phase with mEOS2-CENP-A and fixed them for imaging in the next cell cycle in G1. The clusters of the newly deposited CENP-A (at 7h) are similar to the common CENP-A clusters in early G1, which suggests that the spherical shape likely results from re-organization of the ring itself, see Suppl. Fig. S13.

How does the diameter of the ring changes from early G1 to late G1?

A> This can be seen from Figure 2: on average, the diameter slightly decreases over the time points, see also new Suppl. Fig. S4. Also, the structure becomes more compact, showing a transition towards a denser CENP-A distribution, which may originate from newly synthesized CENP-A (see Fig. 2); we have now clarified this in the manuscript.

3. Understanding the spatial relationship between CENP-A and H3 chromatin has been of high interest to the centromeric field. However, lack of tools has hindered gaining insights into this aspect. STORM co-localization of CENP-A and H3 should reveal how H3 is arranged both linearly and in 3D at centromeric regions. If not the exact placement of H3 alongside CENP-A, authors should at least find out if H3 is present on this ring or not. Or it may reveal if the H3 coats inside or outside of the ring.

A> The exact placement of H3 alongside CENP-A within chromatin is difficult to achieve because of limitations of current microscopy techniques and the denser nature of the chromatin fiber (except for stretched chromatin fibers). To address the question asked, one should in fact analyze H3.3 rather than H3. For this, we now imaged H3.3 with CENP-A in U2OS cells. Given the diffuse distribution of H3.3 it is difficult to conclude whether or not it co-localizes with CENP-A (see Suppl. Fig. S10).

4. Figure 3B – I don't understand the point of showing all the panels. They all look very similar to me. Please explain the differences if any. Figure 3C - A schematic inset for each panel would be helpful to show the progression of the density distribution from a shell to a disc shape.

A> We have changed the colormap to grey to simplify the display. The comparison of the time points shows that the rosette-like structure (left panel) evolves towards a more compact organization in which the central cavity is filled (panel on the right, Fig. 3C).

5. A discussion about the relevance of CENP-A ring in the kinetochore structure and function is completely missing. The authors should discuss what is already known from previous STORM imaging of centromeric proteins. For example, super-resolution images of mitotic centromeres by Wynne and Funabiki (PMID: 27170176) has revealed ring-shaped kinetochores (stained with Ndc80, CENP-E, BubR1) in which inner kinetochore proteins (CENP-A, CENP-C & Mis12) occupy a central position. So the ring shape seems to be a structural feature of the entire kinetochore where the inner kinetochore components including CENP-A might undergo compaction and acquire a spheroid shape. Since CENP-A is the innermost protein in this ring, it would make sense for HJURP to bind and access centromeric chromatin from the inside of the ring. The rosette by itself might not have an exclusive function in CENP-A loading by HJURP so the term assemblosome is premature.

A> While we agree that the organization of the kinetochore structure is an interesting aspect it may go beyond this study. We have nevertheless compared the centromeric CENP-A organization in mitosis (rather than G1 which is the focus of this study), which displays clear elongated rod- or ring-like structures (see Suppl. Fig. S9). These are clearly distinct from the globular rosette-like structures in early G1, during which CENP-A loading nucleates on HJURP. These additional interesting data show that the CENP-A organization is very dynamic and consists of an evolution from a disk-like structure in mitosis to a globular rosette-like structure in G1. We have kept the term "assemblosome" as a hypothesis in the discussion, but removed it elsewhere in the text and abstract.

6. For the amount of information available in the current version of the manuscript, it can be significantly shortened. Line 69 – please replace "thundering".

A> We have streamlined the manuscript and removed the term cited.

Reviewer #2 (Remarks to the Author):

Andronov et al. CENP-A nucleosome clusters form rosette-like structures around HJURP during G1.

In this paper the authors present super-resolution imaging data of CENP-A and HJURP in human cells at different cell stages. They show that the structure of the CENP-A clusters changes will cell cycle and that this is corelated to the presence of HJURP at the centres of these clusters.

Overall this is an interesting paper with potential to significant extend our understanding of the biological function of CENP-A, its recruitment to the centromere, and hence its involvement in chromosome segregation. Broadly the paper is extremely well written with clear descriptions and good figures. The statistics, such as N numbers and SEM versus SD are clearly stated.

A> We thank the reviewer for the very positive feedback. This study indeed provides unprecedented insights into CENP-A function.

I have a few minor comments.

1) The authors show a large number of SMLM images but don't give any indications as to the quality of the data fitting. They should state some information about number of localisations and average localisation precision to give an idea about the real data quality. Alternatively, they could use a technique such as Fourier ring correlation to measure actual image resolution.

A> We have now added a table summarizing information about SMLM data (number of localizations, photon counts and FRC resolution). This is now added in the Methods section (Table 2).

2) In the final section the authors state "This suggests that the newly discovered specific rosette-like structure of the HJURP- centromeric nucleosome complexes is probably a common feature of eukaryotic cells at the G1 phase of the cell cycle." They have examined this structure in two human derived cell lines. It would be interesting to show that this is common feature across many eukaryotes but this paper does not show this.

A> We have now extended the study to comprise a mouse cell line (MEF), where we find similarly shaped clusters of CENP-A (Suppl. Fig. S14).

3) They suggest in the methods section that this is the first application of single particle reconstruction methods from cryoEM to SMLM data. This is not true, eg see DOI: 10.1109/TMI.2018.2795464.

A> The mentioned paper was published recently, when we were already at the final stage of our paper preparation. We have now changed "the first application" to "one of the first applications" and cited the publication.

Overall I think this paper is suitable for publication with minor updates.

Reviewer #3 (Remarks to the Author):

The authors present an interesting study on the distribution of CENP-A in G1. In general the work is good, but not as thorough as I would expect for Nature Communications. There are some reasonably major issues that should be addressed prior to acceptance for publication. There are also a number of spelling and idiomatic inconsistencies (a few of which I've listed at the end of this review) and I'd encourage the authors to thoroughly proof-read their work before submitting a revised version. Starting with my scientific concerns.

A> We thank the referee for the comments which are addressed below.

- When imaging shortly after mitosis it is reasonable to assume that much of the kinetochore is still be intact. From previous EM studies this has been shown to be an incredibly densely packed structure, and one might reasonably expect access by antibodies to be impaired within the centre of the kinetochore. A very plausible explanation of the observations presented here would be if access to the centrally located CENP-A only became possible as the kinetochore disassembles throughout G1. The authors should eliminate this as a possible cause (or at the very least discuss it). The most compelling test for this would be to use a tagged version of CENP-A (e.g. with a SNAP or HALO tag – both of which would allow labelling with STORM compatible dyes, or with a switchable fluorescent protein).

A> We have now imaged CENP-A, tagged with the mEOS2 photo-convertible protein, using transient transfection as well as stable transfection of U2OS cells. PALM imaging of mEOS2 reveals the same hollow shape of CENP-A clusters in early G1 (see Suppl. Fig. S5), as observed with antibody labelling. This shows that the results are not related with accessibility issues of the antibodies.

- Previous (mainly EM, but also confocal measurements based on the fact that CENP-A typically presents as a spot which is a little bit larger than the diffraction limit) work has established that the centromeric regions have a size of ~200-500nm, and that there is a lot of variation between chromosomes within one cell. The amount of variation that is known to exist in the structure makes any class averaging efforts extremely tenuous. As it stands there is very little justification for using the alignment and back projection methods used to obtain the data in Sup. Fig 3, which are based on the assumption that each of the structures being averaged is essentially identical. Critically the conclusions do not depend on this data – the 2D and 3D dSTORM images provide

reasonably compelling evidence for reduced labelling towards the centre of the centromeres. Rather I feel that it's inclusion reduces my confidence in the rest of the work (if the authors are happy to apply an analysis method for which the fundamental assumptions are not met, can I have any confidence in the rest of their data?).

A> These worries are based on the assumption suggested by previous studies that there would be significant size variations for a given time point, which is in fact not the case. This is to our knowledge the first study in G1 using SMLM rather than diffraction-limited imaging that reveals CENP-A clusters. We now performed a size analysis (see Suppl. Fig. S4 and methods section) that shows that different centromeres have a similar shape and size in G1-synchronized cells (this can also be seen from the figures in our paper, e.g. Fig. 1B). Therefore, our analysis based on back-projection and 3D reconstruction is well founded. Indeed, and remarkably, the cluster structures show an overall similar organization within a given cell, which is consistently observed regardless of the technique (dSTORM, PALM or STED) or the cell type (U2OS, HeLa or MEF).

- My class averaging concerns also apply (to a considerably lesser extent) to the rotationally averaged data in Fig 3. If object size varies, these become an imperfect tool. In this case, however, it is probably good enough. That said a quantification of the distribution of CENP-A cluster sizes at each time point would be a useful control. A broadening in the distribution of radii of an annular structure would also lead to a filling in of the centre of a rotational average and this quantification should be able to put a limit on the magnitude of such an effect.

A> As described above, there are no significant size variations for a given time point.
Over time, the diameter also remains rather constant (as quantified in Fig. 3, panel C and visible in panel B, the variance of the size during averaging is low, see new Suppl. Fig. S4) and it is the interior structure that changes over time to become denser inside. Obviously, rotational averaging of all clusters within a given cell discards differences between individual clusters, but it allows to see a global trend in the cluster shape as a cell progresses through the cell cycle, which is quantified here and made better visible now in the modified Fig. 2.

- The authors assert that HJURP localizes to the centre of the CENP-A clusters, but do not present any quantitative evidence to support this (Fig 4 is suggestive, but not at all quantitative). A simple solution would be to also calculate the rotational average of HJURP (using the centres inferred for CENP-A) and to plot both radial profiles in the same graph.

A> We have addressed this point by calculating radial distributions of the localization of HJURP and CENP-A, confirming the analysis presented (see new panels C&D in Fig. 4).

- Previous studies have suggested that the kinetochore (and by extension the centromeric chromatin) are a flat, disc-like structure. It would be interesting to discuss the results in this context, and more generally in the context of previous EM studies. How much of the axial extent of the stuctures in Fig 2 is due to the axial localization precision and/or systematic errors?

A> To our knowledge, all previous studies focused on kinetochore or on the centromeric chromatin in mitosis, where the disk-like structure is observed. In our study, however, we focus on interphase, and we found no evidence that interphase centromeres are flat structures. If it was so, a significant part of the CENP-A clusters would have a rod-like or an oval shape in 2D, which we do not observe at all. In new additional experiments, we have compared the centromeric CENP-A organization in mitosis (rather than G1 which is the focus of this study), which displays clear elongated rod- or ring-like structures compatible with the disc-like structures shown in previous studies (see Suppl. Fig. S9).

- The authors talk about numbers of CENP-A (L 144) specifying that the clusters contained several hundred CENP-A, but do not detail how they got these numbers. Counting molecules in dSTORM (and other localization microscopies) is not simple, particularly when combined with antibody labelling, and any attempt to count molecules need to be described in detail. The authors should either describe in detail how they arrived at the count, complete with the relevant calibration measurements, or remove this detail from the paper (as the conclusions do not depend on it).

A> This number was just the number of fluorophore localizations detected at a centromere, which is not the number of CENP-A molecules, but can be proportional to it; therefore, the value is meaningful only as a relative measure, as the referee suggested (see below). Since now we did counting of CENP-A molecules at different time points, we have indicated relative amounts (see below).

- A useful addition would be a characterization of the relative amounts of CENP-A in a cluster at

each time point (this is much easier than absolute molecule counts). The authors stress the fact that newly synthesized CENP-A is being incorporated and a quantification of the relative amounts would help this story (you would expect roughly 2x as many localizations from clusters towards the end of the imaging period, whereas a naïve examination of the example structures in Fig 3A would suggest that this more than doubles – potentially supporting for the accessibility argument.).

A> Because with antibody labelling there are some unknown variables, which may affect the number of SM localizations of a labelled protein, we now did a new additional experiment using a cell line stably expressing CENP-A-mEOS2. We observe a significant increase of the number of localizations from 1.5 to 7 hours after mitosis (~1.5 times), which is consistent with the idea that the CENP-A concentration should double during G1 as the referee suggested (Suppl. Fig. S3).

Minor points

Fig 3: consider using an alternative colour map (e.g. greyscale) for the rotational averages. The "jet" color map is a poor choice for representing changes in density, as the brightest area appears darker than much of the rest of the images

A> Done.

L 69 – "thundering importance" – not idiomatic

A> Removed.

L 79 – "down to" implies a lower bound, not a range

A> Rephrased.

L 88 – "peculiar"- this implies abnormal – "special" might be more idiomatic

A> Done.

L 93 – spelling – "Alexa Fluor"

A> Done.

L 98 – "using adaptive optics" – clarified in methods, but unclear what adaptive optics is used for (generating the astigmatism) at this point. Would be clearer if omitted here and just left in

methods.

A> Rephrased.

L148/149 – "colocalization analysis" – This would usually imply some form of quantification of the spatial relationship between the two labels. You just present two colour images, and don't present the results of any colocalization analysis.

A> Quantification is done now (Fig. 4C-D).

L 367 – "exposure" time (not exposition)

A> Done.

L 389 – events

A> Done.

L 391 – single molecule return (not returns)

A> Done.

L407 – molecule chaining – also need to specify time window (i.e. how many frames can a molecule be dark and still be linked and averaged)?

A> In fact we didn't limit the maximal time of appearance (i.e., we averaged localizations on all consecutive frames until there was at least one empty frame within the radius of 50 nm). This is now clarified in the methods section.

Reviewers' comments:

Reviewer #1 (Remarks to the Author):

The authors have adequately addressed my concerns.

Reviewer #2 (Remarks to the Author):

Andronov et al. CENP-A nucleosome clusters form rosette-like structures around HJURP during G1.

In this paper the authors present super-resolution imaging data of CENP-A and HJURP in human cells at different cell stages. They show that the structure of the CENP-A clusters changes with cell cycle and that this is corelated to the presence of HJURP at the centres of these clusters.

In this resubmission the authors have also added imaging in mouse cells to demonstrate that the effects they see are generally applicable. With reference to my previous review and the updates of the paper relating to it.

1) The authors have added a table summarizing information about the SMLM data quality however this raises some questions of its own. They quote average localizations per cell for various conditions but don't say anything about N number or variance in these numbers. There are strange discrepancies in the stated resolutions from FRC and the median photon counts. For instance, the final two rows are DNA-YoYo-1 and H3.3 Alexa-488, both with 1200 median photon counts, however the DNA has a FRC resolution of 120nm while the H3.3 has 65 nm.

2) I still feel their claim about the generality of this "conserved functional role" is too strong given the fact they have now expanded it from just humans, to humans and mice. The phrase "…as well as across different eukaryotes…", although technically correct is still overstating their case.

Other points:

The authors image analysis methods are not entirely clear.

1) Line 329 states "The images of clusters were then normalized…". How?
2) "the resulting aligned particles were again rotationally averaged and their radial profile was calculated in Matlab". I am entirely unclear if the authors used the synthetic images, or the drift corrected raw localization positions for this.
3) It is not clear if the authors made any effort to account for background in their localisations. This can significantly affect radial averages since a background with a constant average density adds many more localizations further from the centre of the radial average due to the much larger area. However, this appears to have been done for the following section on mEOS and HJURP.
4) The methods section seems to gloss over many important issues, such as correction for chromatic aberrations and how consecutive localizations were assessed to be merged. Some of the paper relies upon exact alignment of two colour SMLM images yet the method for alignment of these two image sets is not described at all.
5) They sate that they were "using several different cells at the same time point" (line 363) but don't clarify if they made any attempt to produce experimental or biological repeats in order to reduce the likely hood of some extraneous systematic effect impacting their data. The lack of this information suggests that all images were from a single repeat and indeed only using one sample.

I still feel that this is an interesting paper worthy of publication but I think that the methods section needs to be revised to make clearer exactly what analysis was done and how.

Reviewer #3 (Remarks to the Author):

The revised version of the paper has been improved and addresses some of my concerns, e.g. the inclusion of radial profiles of HJURP, and the mEOS control which provides supportive evidence against an accessibility hypothesis.

I remain singularly unconvinced, however, on the appropriateness of 3D average reconstruction based on projections. In this respect, the response to reviewers is borderline deceptive. In an attempt to justify the averaging, the authors state that they have quantified the distribution of centrosome sizes, citing Sup. Fig. 4. The data presented in Sup Fig 4 does not present this quantification, but rather a comparison of the mean cluster size (of already averaged clusters) across different time points. This tells us nothing about the spread of cluster sizes within one time point. The two control measurements I had requested and which would actually be somewhat informative were a) distribution of cluster sizes within one cell / time point and b) variability in cluster sizes as a function of time (i.e. how the std. deviation of a) changes). As a minor aside, it is unclear what the error bars in Sup Fig 4 represent - is this variation between cells? and is it std. deviation or std. error?

The one remaining justification presented is that the images in Fig 1B look to be the same shape and size, which is a very weak justification (my personal read would be that they look sufficiently different that projection averaging would be unlikely to give reliable results). Remembering that even in EM where there are good reasons to assume that a protein will have a consistent structure, this type of averaging becomes controversial and error prone when there are different conformational states present. There are very, very few macromolecular complexes in the cell that lend themselves to particle averaging on the PALM/STORM length scale, the nuclear pore complex being a notable exception due to it's extreme reproducibility (although even then it's somewhat contentious - high SNR, non-averaged, STORM images show that the 8-fold symmetry is sometimes broken with occasional 9-membered rings). To assume that a dynamic structure (one of the papers claims) which roughly doubles in protein count during the cell cycle, is a suitable target for particle averaging is obtuse.

The reason I feel strongly about this is that, with the exception of the particle averaging I think this is a good paper. I believe the conclusion that clusters have a hollow centre in G1 is correct. The reconstruction, however, rail-roads the reader into assuming a barrel geometry, and I don't believe that the data is sufficient to support this. A hollow sphere, for example, would look very similar in the projections, the radial profiles, and also in the 3D localisation data (due to both limitations in effective axial localisation range and the effect of poorer axial resolution on the density reconstruction)

Point-to-point response to the Reviewers' comments:

Reviewer #1 (Remarks to the Author):

The authors have adequately addressed my concerns.
We thank the referee for the final evaluation.

Reviewer #2 (Remarks to the Author):

Andronov et al. CENP-A nucleosome clusters form rosette-like structures around HJURP during G1.

In this paper the authors present super-resolution imaging data of CENP-A and HJURP in human cells at different cell stages. They show that the structure of the CENP-A clusters changes with cell cycle and that this is corelated to the presence of HJURP at the centres of these clusters.

In this resubmission the authors have also added imaging in mouse cells to demonstrate that the effects they see are generally applicable. With reference to my previous review and the updates of the paper relating to it.

Our revised manuscript indeed addressed all the points originally raised and contains a large amount of additional experimental data which clarify and further strengthen our original analysis.

1) The authors have added a table summarizing information about the SMLM data quality however this raises some questions of its own. They quote average localizations per cell for various conditions but don't say anything about N number or variance in these numbers. There are strange discrepancies in the stated resolutions from FRC and the median photon counts. For instance, the final two rows are DNA-YoYo-1 and H3.3 Alexa-488, both with 1200 median photon counts, however the DNA has a FRC resolution of 120nm while the H3.3 has 65 nm.

It is known that real resolution of SMLM data does not only depend on photon counts. The YoYo-1 dye was not especially well performing for our SMLM imaging, namely it did not blink well, and this is why the estimated FRC resolution is lower as compared to better performing Alexa dyes. Lower resolution of DNA imaging, however, does not affect any conclusions of our paper, as it was used only to show that the chromatin is decondensed (Suppl. Fig. S11), which does not require high resolution. We have clarified this now in the methods section and indicated N numbers of experiments and cells in Table 2 as suggested by the referee.

2) I still feel their claim about the generality of this "conserved functional role" is too strong given the fact they have now expanded it from just humans, to humans and mice. The phrase "…as well as across different eukaryotes…", although technically correct is still overstating their case.

We have removed this phrase now.

Other points:

The authors image analysis methods are not entirely clear.

1) Line 329 states "The images of clusters were then normalized…". How?

Normalization was done by taking into account the standard deviation of pixel values, as now indicated in the methods section.

2) "the resulting aligned particles were again rotationally averaged and their radial profile was calculated in Matlab". I am entirely unclear if the authors used the synthetic images, or the drift corrected raw localization positions for this.

This uses the reconstructed super-resolution images (after drift-correction), this is now specified in the methods section.

3) It is not clear if the authors made any effort to account for background in their localisations. This can significantly affect radial averages since a background with a constant average density adds many more localizations further from the centre of the radial average due to the much larger area. However, this appears to have been done for the following section on mEOS and HJURP.

See answer above, since reconstructed super-resolution images were used there is no such problem here. Rotation averaging of images with uniformly distributed noise gives an image with a uniform distribution of intensity, therefore such a correction is not needed for rotational averaging of images. For the localization-based analysis (see data for mEOS2 and HJURP) radial weighting correction was indeed done, as described in the methods section. Note that Voronoi diagram based image reconstruction and segmentation also reduces background effects (see Andronov et al., 2016 & 2018).

4) The methods section seems to gloss over many important issues, such as correction for chromatic aberrations and how consecutive localizations were assessed to be merged. Some of the paper relies upon exact alignment of two colour SMLM images yet the method for alignment of these two image sets is not described at all.

Correction of chromatic aberrations and merging of consecutive localizations was done in our software SharpViSu (Andronov et al., 2016). Chromatic aberrations were first calibrated using Tetraspeck beads, the shift between the channels was interpolated with a 2d-order polynomial and was subtracted from the localizations of one of the imaging channels. Consecutive localizations were addressed in the following way: for each original localization N on frame n, localizations within a distance of 50 nm from N on frame n+1, were searched for. If found, the search continued for the frame n+2, etc. The search stopped when there was no localization on the following frame. All the found localizations were merged into a single one, which was assigned to the frame n, its coordinates were set as the average of the coordinates of all the consecutive localizations and the photon count was set as the sum of the photon counts. This is now specified in the methods section.

5) They sate that they were "using several different cells at the same time point" (line 363) but don't

clarify if they made any attempt to produce experimental or biological repeats in order to reduce the likely hood of some extraneous systematic effect impacting their data. The lack of this information suggests that all images were from a single repeat and indeed only using one sample.

Experiments that constitute the main results of the paper (imaging of CENP-A alone and with HJURP in synchronized cells) were repeated several times using new seed cells each time (e.g. results presented in Fig. 2 and 4 are from different cells, different antibodies and image acquisitions but give the same distribution for CENP-A). Samples for STED imaging were also prepared independently from the samples for SMLM. We also used different secondary antibodies for different repeats to eliminate the impact of different SMLM performance of different dyes (summarized in Table. 2). In addition to stable transfection of mEOS2-CENP-A, an independent transient transfection was also done, revealing a similar shape of clusters. This is now added to Table 2.

For cluster analysis data were processed for single cells in which the centromeric regions are very similar between each other (synchronization cannot be done to the level of having all cells in exactly the same state). For radial profile analysis and time point analysis many cells were used, which underlines the significance of the analysis. This is now clarified in the methods section.

I still feel that this is an interesting paper worthy of publication but I think that the methods section needs to be revised to make clearer exactly what analysis was done and how.

We appreciate the final comments and agree that this is an interesting study that in fact provides many new insights into CENP-A function during G1 phase. We have now clarified all points in the methods section.

Reviewer #3 (Remarks to the Author):

The revised version of the paper has been improved and addresses some of my concerns, e.g. the inclusion of radial profiles of HJURP, and the mEOS control which provides supportive evidence against an accessibility hypothesis.

Indeed, our manuscript includes a series of additional data that clarify and strengthen our original manuscript.

I remain singularly unconvinced, however, on the appropriateness of 3D average reconstruction based on projections. In this respect, the response to reviewers is borderline deceptive. In an attempt to justify the averaging, the authors state that they have quantified the distribution of centrosome sizes, citing Sup. Fig. 4. The data presented in Sup Fig 4 does not present this quantification, but rather a comparison of the mean cluster size (of already averaged clusters) across different time points. This tells us nothing about the spread of cluster sizes within one time point. The two control measurements I had requested and which would actually be somewhat informative were a) distribution of cluster sizes within one cell / time point and b) variability in cluster sizes as a function of time (i.e. how the std. deviation of a) changes). As a minor aside, it is unclear what the error bars in Sup Fig 4 represent - is this variation between cells? and is it std. deviation or std. error?

The mean cluster size analysis in Suppl. Fig. S4 shows that the sizes are very similar (for one given time point, not across different time points), hence the distribution of centromere sizes is small. Therefore, different 2D projections can be averaged into a 3D reconstruction. Note also that the method of common lines that we used provides an angular reconstitution directly from the data, without using any references that could otherwise introduce a bias. Hence, the fact that a 3D reconstruction can be obtained from a common line approach also underlines the fact that the objects are structurally similar (at least to a given resolution level, see answer to the next question), otherwise the method would not work. Thus there is no reason to not be convinced by the appropriateness of this analysis. Please note that re-projections of the obtained 3D reconstruction correspond well to the input particles (Suppl. Fig. S6), as described in the methods section.
The error bars in Suppl. Fig. S4 represent the standard deviation between individual centromeres (between all centromeres from several cells synchronized at a given time point), therefore it already answers the referee's question. The analysis of cluster sizes was done for each individual time point, as suggested (Suppl. Fig. S4). This was clarified in the Suppl. Fig. S4 legend.

The one remaining justification presented is that the images in Fig 1B look to be the same shape and size, which is a very weak justification (my personal read would be that they look sufficiently different that projection averaging would be unlikely to give reliable results). Remembering that even in EM where there are good reasons to assume that a protein will have a consistent structure, this type of averaging becomes controversial and error prone when there are different conformational states present. There are very, very few macromolecular complexes in the cell that lend themselves to particle averaging on the PALM/STORM length scale, the nuclear pore complex being a notable exception due to it's extreme reproducibility (although even then it's somewhat contentious - high SNR, non-averaged, STORM images show that the 8-fold symmetry is sometimes broken with occasional 9-membered rings). To assume that a dynamic structure (one of the papers claims) which roughly doubles in protein count during the cell cycle, is a suitable target for particle averaging is obtuse.

The images in fact look rather similar, this is why a 3D reconstruction could be envisioned in the first place. Differences can be seen in the finer details of the clusters, and these would be averaged out in the 3D reconstruction process. This is normal and would simply limit the resolution of the reconstruction, unless structural heterogeneity would be addressed in more detail by sorting of structures (as we are well aware of as we have done many developments in that area in cryo-EM, see Klaholz et al., Nature 2004; Simonetti et al., Nature 2008; Klaholz 2015; von Loeffelholz, COSB 2017 etc.). But the main point is that a common sub-structure exists in all the CENP-A clusters, which is confirmed also by direct 3D SMLM analysis and thus is fully consistent (Fig. 3). The description of this particular structure applies to early G1 where a defined structural state is observed (clarified now in the methods section). The method was not applied to later time points because the structure differs and evolves, which is one of the points of the paper. Finally, doubling of protein counts during the cell cycle comes from the incorporation of new CENP-A protein as discussed in the discussion part of the manuscript.

The reason I feel strongly about this is that, with the exception of the particle averaging I think this is a good paper. I believe the conclusion that clusters have a hollow centre in G1 is correct. The reconstruction, however, rail-roads the reader into assuming a barrel geometry, and I don't believe that the data is sufficient to support this. A hollow sphere, for example, would look very similar in the

projections, the radial profiles, and also in the 3D localisation data (due to both limitations in effective axial localisation range and the effect of poorer axial resolution on the density reconstruction).

We thank the referee for the final comments. We agree that the structure in fact looks like a barrel. In 2D projection images these may look differently, this is why the hole in the middle is not visible in all particle images or object orientations (see Fig. 3B). However, our 3D SMLM analysis supports the idea that the underlying structure is barrel-like, which is also consistent with the particle averaging approach. We have rephrased this in the manuscript. Distortion effects should be relatively small because the effective axial localization precision was made more isotropic by using a higher astigmatisms during 3D SMLM; this is now described in more detail in the methods section.

REVIEWERS' COMMENTS:

Reviewer #2 (Remarks to the Author):

The authors have adequately addressed the points made in my previous reviews.

Reviewer #3 (Remarks to the Author):

I can see that the authors and I are not going to agree on the particle averaging and 3D reconstruction. I do not believe that my concerns have been adequately addressed and it remains my opinion that the reconstruction is not appropriate and will likely be erroneous. As a way forward, I'd suggest the following.

a) The authors provide a histogram of the angles / orientations assigned to particles in the reconstruction. This is a relatively weak line of evidence, but one would expect individual centromeres to be randomly oriented in space which would give rise to a uniform distribution of assigned angles. My hypothesis that the barrel shaped reconstruction could be the result of a spherical shape combined with known limitations in PALM/STORM imaging would likely give rise to a non-uniform distribution of centromere orientations (biased towards the open ends of the barrel orientating vertically).

b) The editor asks another reviewer to comment specifically on the appropriateness of the 3D averaged reconstruction.

I do not need to see the manuscript again.

Point-to-point response to referees:

Reviewer #2 (Remarks to the Author):

The authors have adequately addressed the points made in my previous reviews.

Reviewer #3 (Remarks to the Author):

I can see that the authors and I are not going to agree on the particle averaging and 3D reconstruction. I do not believe that my concerns have been adequately addressed and it remains my opinion that the reconstruction is not appropriate and will likely be erroneous. As a way forward, I'd suggest the following.

a) The authors provide a histogram of the angles / orientations assigned to particles in the reconstruction. This is a relatively weak line of evidence, but one would expect individual centromeres to be randomly oriented in space which would give rise to a uniform distribution of assigned angles. My hypothesis that the barrel shaped reconstruction could be the result of a spherical shape combined with known limitations in PALM/STORM imaging would likely give rise to a non-uniform distribution of centromere orientations (biased towards the open ends of the barrel orientating vertically).

b) The editor asks another reviewer to comment specifically on the appropriateness of the 3D averaged reconstruction.

I do not need to see the manuscript again.

The overall structure is relatively globular, not barrel-like as the referee suggested earlier and we then first agreed on; the reconstruction in fact shows a spherical shape with a cavity in the center which resembles a rosette. The Euler plot that we provide in the Supplementary Information shows a good angular distribution without preferential orientations, consistent with the observation of a spherical shape rather than an elongated barrel-like object. Please note that this 3D reconstruction obtained from 2D SMLM imaging is consistent with the 3D structure obtained by direct 3D SMLM imaging (in which the effective axial localization precision was made more isotropic by using a higher astigmatisms during 3D SMLM to avoid distortion effects). The absence of preferred orientations as seen in the Euler plot also indicates that imaging was not a limiting factor in terms of effective axial localization range or potential poorer axial resolution, which we agree can be a general issue in imaging. If present, these effects would be averaged out in the 3D reconstruction due to the fact that the individual objects indeed have different orientations in the sample when being imaged. This is discussed in the Supplementary Information.
